# Dietary nitrogen alters codon bias and genome composition in parasitic microorganisms

**DOI:** 10.1186/s13059-016-1087-9

**Published:** 2016-11-15

**Authors:** Emily A. Seward, Steven Kelly

**Affiliations:** Department of Plant Sciences, University of Oxford, South Parks Road, Oxford, OX1 3RB UK

**Keywords:** Genome evolution, Mutation bias, Elemental selection, Nitrogen metabolism, Synonymous codon use, Comparative genomics, Codon bias, Kinetoplastids, Mollicutes, Stoichiogenomics

## Abstract

**Background:**

Genomes are composed of long strings of nucleotide monomers (A, C, G and T) that are either scavenged from the organism’s environment or built from metabolic precursors. The biosynthesis of each nucleotide differs in atomic requirements with different nucleotides requiring different quantities of nitrogen atoms. However, the impact of the relative availability of dietary nitrogen on genome composition and codon bias is poorly understood.

**Results:**

Here we show that differential nitrogen availability, due to differences in environment and dietary inputs, is a major determinant of genome nucleotide composition and synonymous codon use in both bacterial and eukaryotic microorganisms. Specifically, low nitrogen availability species use nucleotides that require fewer nitrogen atoms to encode the same genes compared to high nitrogen availability species. Furthermore, we provide a novel selection-mutation framework for the evaluation of the impact of metabolism on gene sequence evolution and show that it is possible to predict the metabolic inputs of related organisms from an analysis of the raw nucleotide sequence of their genes.

**Conclusions:**

Taken together, these results reveal a previously hidden relationship between cellular metabolism and genome evolution and provide new insight into how genome sequence evolution can be influenced by adaptation to different diets and environments.

**Electronic supplementary material:**

The online version of this article (doi:10.1186/s13059-016-1087-9) contains supplementary material, which is available to authorized users.

## Background

Cells are primarily composed of a few major macromolecules (proteins, RNA, DNA, phospholipids and polysaccharides) that are constructed from monomers (amino acids, nucleotides, etc.). The sequence of these monomers is important for correct molecular function, although there is often flexibility allowing for monomer usage bias. For example, synonymous codons specify the same amino acid and different nucleotide sequences can thus code for the same polypeptide. Multiple competing factors have been proposed to bias the relative use of synonymous codons. These include, but are not limited to, neutral drift (as a result of mutational biases during DNA replication and repair) [1–3], iso-accepting tRNAs [4], translational efficiency and accuracy [5–8], altered gene splicing and protein folding [9], mRNA purine loading as a result of temperature [10, 11] and generation time [12]. Furthermore, multiple factors such as UV radiation, nitrogen fixation and parasitism have been proposed to explain GC variation in prokaryotes [13, 14]. However, the impact of monomer availability (i.e. the relative availability of different nucleotides within the cell) on codon bias has been largely unexplored. We propose that differences in dietary nitrogen should cause concomitant differences in codon bias between closely related organisms whose similar lifestyles exclude alternative explanations.

Though the impact of monomer availability on synonymous codon use has yet to be elucidated, several studies have investigated the elemental composition of macromolecules (protein, DNA, RNA, etc.) using genomics data and bioinformatics tools [15]. Pioneering work in this area focused on protein evolution and demonstrated that as well as the energetic costs associated with synthesising each monomer (amino acid), the monomer’s elemental demands can bias usage in nutrient-limiting environments [16]. Here it was shown in *Escherichia coli* and *Saccharomyces cerevisiae* that enzymes required for metabolic processing of an element have reduced quantities of that element in their sequences [16]. Similar studies in plants have shown that there was a 7.1 % reduction in nitrogen use in amino acid side chains when plant proteins were compared to animal proteins [17]. It was proposed that this reduction was due to differences in the relative nitrogen availability of these two groups of organisms as plants are nitrogen limited in comparison to animals [17]. More generally it has also been seen that there is a negative correlation between protein abundance and the atomic requirements of its constituent monomers [18].

Elemental limitation also has an impact on genetic sequences (DNA and RNA), which are composed of nucleotides that are either scavenged from the organism’s environment or built from metabolic by-products. Like amino acids, the biosynthesis of each nucleotide differs in energetic and atomic requirements, with GC pairs consuming more ATP and requiring more nitrogen for biosynthesis than AT pairs [19]. The differences in energetic cost have been proposed to cause differences in the relative abundance of nucleotides within the cell, ultimately leading to nucleotide usage bias in genomic sequences [19]. In support of this hypothesis, it has been shown that imbalances in the relative availability of nucleotides within a cell or restrictions in nucleotide biosynthesis can lead to mutational biases that alter genome nucleotide content [15, 20]. Such differences are manifested as usage biases in organisms that have evolved in conditions where there is a persistent elemental limitation. For example, domesticated crops, which have been cultivated with nitrogen fertilisation for thousands of years, and nitrogen-fixing plants show increased use of nitrogen-rich nucleotides in the transcribed strand of their intergenic regions compared to wild plants, which are relatively nitrogen limited [21]. Furthermore, both protein elemental sparing and codon usage bias have been seen in 148 bacterial species, with significant correlations between carbon and sulfur usage and adaptive codon usage bias [22].

Given that changes in metabolism can lead to changes in the relative abundance of nucleotides, it follows that changes in an organism’s diet (the sum of all food consumed by an organism) could have the potential to alter the nucleotide composition of the genome. Specifically, as nucleotides contain different numbers of nitrogen atoms (A/G = 5, C = 3, T/U = 2), differences in dietary nitrogen content should result in concomitant differences in the relative abundance of nucleotides within the cell and thus differences in nucleotide use between species. Moreover, these differences in nucleotide use should be detectable by comparing the nucleotide sequences for orthologous protein-coding genes in organisms that share a common ancestor but have since adapted to utilise different dietary inputs. Here redundancy in the genetic code would allow differences in nucleotide use between species to manifest as changes to nucleotide sequences without necessarily altering the encoded amino acid sequence.

Microbial parasites represent an ideal model system to investigate this phenomenon and determine the effects that changes in dietary input have on the evolution and composition of genome sequences. This is because microbial parasites typically have streamlined metabolisms and often obtain energy from catabolism of a limited set of host biomolecules. Furthermore, closely related parasites often utilise different metabolic strategies and obtain energy from catabolism of different host-derived compounds and even related parasites that colonise the same host niche can obtain energy from catabolism of different inputs [23]. Thus, comparative genomics between parasites that share a common ancestor but have adapted to utilise different host-derived biomolecules has the potential to reveal the effects of changes in diet on the evolution and composition of genome sequences.

Here we provide a global analysis of gene sequence evolution associated with adaptation to changes in diet. We show in two monophyletic groups of parasites (one eukaryotic and one bacterial) that adaptation to diets with differing nitrogen content produces a concomitant effect on nucleotide compositions (and hence nitrogen content) of orthologous RNA sequences. Those parasites that have adapted to low nitrogen content diets have low nitrogen content sequences while those parasites that have adapted to high nitrogen content diets have high nitrogen content sequences. We construct a novel model for synonymous codon use that is sufficient to explain the genome-wide usage of synonymous codons with >90 % accuracy. We show that this model used in a predictive capacity is able to identify the metabolic capacity of related parasites from raw nucleotide sequences. Taken together our findings provide significant new insight into the relationship between diet, metabolism and genome evolution and provide a novel mechanistic explanation for genome-wide patterns of synonymous codon use.

## Results

### Choice of model organisms and inference of orthogroups

**Fig. 1:**
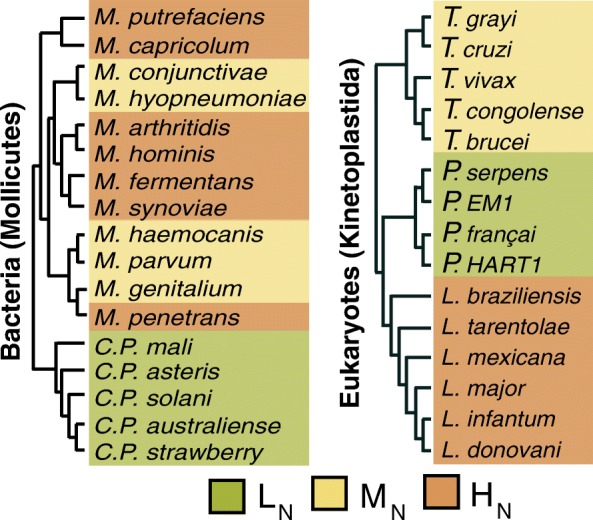
Phylogenetic trees of the parasites used in this study shaded according to their host and metabolic strategy. *Green* indicates plant parasites that obtain energy from carbohydrate catabolism (low nitrogen availability, *L*_*N*_). *Yellow* indicates animal parasites that obtain energy from carbohydrate catabolism (medium nitrogen availability, *M*_*N*_). *Orange* indicates animal parasites that obtain energy from amino acid and/or amino sugar catabolism (high nitrogen availability, *H*_*N*_)

To test the hypothesis that differences in diet between organisms can impact on the nucleotide composition of their genomes, a comparative genomic analysis was performed using bacterial (Mollicutes) and eukaryotic (Kinetoplastida) parasites that have adapted to different host niches (Fig. 1; Additional file 1: Figures S1 and S2). These parasites were chosen for analysis because none of the species fix nitrogen and so require nitrogenous compounds obtained from their environment [24, 25]. Furthermore, unlike opportunistic parasites or free-living organisms, these parasites are restricted to host niches that differ in the relative abundance of biologically available nitrogen. Specifically, parasites that colonise plant hosts are nitrogen limited in comparison to those that colonise animal hosts [21]. Additionally, the parasites’ pathways for ATP generation differ in liberation of biologically available nitrogen (Additional file 1: Figure S2) [23, 26–29]. The parasites that obtain energy through glycolysis obtain carbon skeletons and re-generate ATP, whereas the parasites that obtain energy through catabolism of arginine or amino sugars additionally obtain biologically available nitrogen (Additional file 1: Figure S2). Thus, the parasites were categorised into three groups depending on host type and whether their metabolism liberates nitrogen. Low nitrogen availability (L_N_) parasites colonise plants and obtain energy through carbohydrate catabolism, medium nitrogen availability (M_N_) parasites colonise animals and primarily catabolise carbohydrates, and high nitrogen availability (H_N_) parasites colonise animals and obtain energy through amino acid or amino sugar catabolism. For further details on the metabolic properties of these parasites see Additional file 2.

A set of orthologous gene groups (orthogroups) covering 15 kinetoplastid genomes (Fig. 1; Additional file 3: Table S1) and an independent set of orthogroups covering 17 Mollicute genomes (Fig. 1; Additional file 3: Table S1) were inferred. Both sets of orthogroups were subject to filtering such that orthogroups were retained for further analysis only if the orthogroup comprised a single-copy gene present in at least three species from each nitrogen availability group (i.e. three L_N_, three M_N_ and three H_N_ species). In this analysis, use of orthologous protein-coding genes allows direct investigation of the effect of adaptation to different metabolic strategies on nucleotide sequences that are derived from a common ancestral state. These genes may also be considered house-keeping genes as the organisms have only one tissue (unicellular) and these genes are conserved across all three groups. The same analysis cannot be done in intergenic regions where ambiguity of orthology prevents paired comparison of sites. Moreover, in the case of bacteria there are too few intergenic regions for robust statistical analyses. Of the 9526 orthogroups identified in kinetoplastids, 3003 satisfied the filtration criteria, encompassing ~40 % of all single-copy genes in these organisms. Similarly, of the 1280 orthogroups identified in the Mollicutes, 168 satisfy the filtration criteria, encompassing 28 % of all single-copy genes in these organisms.

### Low nitrogen availability parasites have low nitrogen content sequences and vice versa

**Fig. 2:**
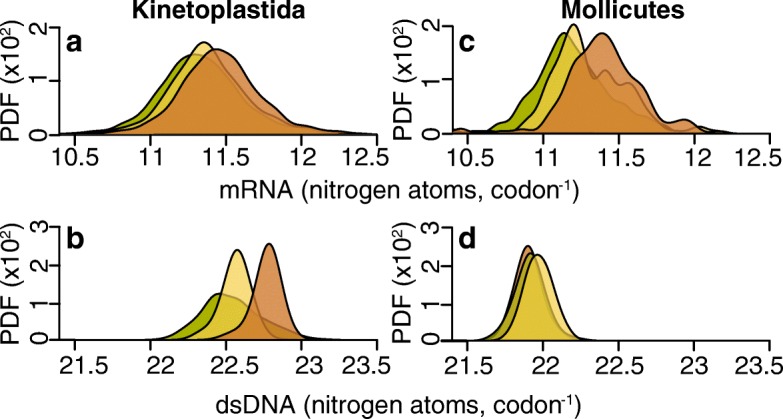
Nitrogen availability influences gene sequences. **a** The average mRNA nitrogen content per codon for 3003 orthologous genes in the Kinetoplastida. **b** The average nitrogen content per double stranded codon (*dsDNA*) for the same genes. **c** As in **a** but for 168 orthologous Mollicute genes. **d** As in **b** but for the Mollicutes. The *y-axis* is the probability density function (*PDF*) for the distributions

In the kinetoplastid parasites 878,193 orthologous codons in the 3003 conserved single-copy orthologous genes were compared. This revealed a significant difference in the nitrogen content of mRNA between the different nitrogen availability groups (Fig. 2a). On average the mRNAs in L_N_ parasites cost one fewer nitrogen atom for every 15 codons compared to the same mRNAs in M_N_ (*p* < 0.001) and one for every seven codons compared to H_N_ (*p* < 0.001). This corresponds to nitrogen savings of ~0.6 % and ~1.3 %, respectively. Given a kinetoplastid cell has ~61,000 transcripts [30] with an average length of 630 codons, L_N_ kinetoplastid parasites would use ~5.5 × 10^6^ fewer nitrogen atoms than H_N_ parasites to produce the same transcriptome. This is enough nitrogen atoms to make ~8700 average sized proteins. The kinetoplastids also exhibit an analogous difference in the nitrogen content of double-stranded DNA (dsDNA; Fig. 2b). Here genes in L_N_ parasites cost one less nitrogen atom for every four codons compared to H_N_, saving roughly 157 nitrogen atoms per gene (~1.1 %). Considering that kinetoplastids are diploid with an average of 8000 genes, this difference in nitrogen cost means that L_N_ parasites use ~2.5 × 10^6^ fewer nitrogen atoms to encode the exact same cohort of genes.

A similar phenomenon was observed when comparing the mRNA sequences in Mollicutes parasites (Fig. 2c). Here comparison of 38,255 orthologous codons in 168 orthologous genes revealed that L_N_ parasites used one fewer nitrogen atom for every nine codons compared to the same mRNAs in M_N_ (*p* < 0.001) and one for every five codons compared to H_N_ (*p* < 0.001). This corresponds to nitrogen savings of ~1 % and ~1.8 %, respectively. Though the Mollicutes exhibit a nitrogen-dependent effect in their mRNAs, the same strong effect is not seen in their dsDNA (*p* = 0.025 when comparing L_N_ and H_N_, *p* < 0.001 comparing M_N_ with either L_N_ or H_N_; Fig. 2d). We propose that the absence of a clear nitrogen-dependent effect at the DNA level is due to a strong GC to AT mutation bias thought to be caused by a lack of dUTPase coupled with a reduced ability to correct erroneous dUTP incorporation in DNA [31, 32]. Thus, though the mRNA for the same genes has a lower nitrogen cost in nitrogen-limited species, the high AT nucleotide composition of the DNA reflects the mutational bias imposed by the lack of dUTPase.

For both the kinetoplastids and Mollicutes an analogous difference is also seen in the nitrogen content of the amino acid side chains of these orthologous sites. The L_N_ parasites use amino acids whose side chains require less nitrogen than the M_N_ and H_N_ parasites (Additional file 1: Figure S3). The slight discrepancy between the M_N_ and H_N_ parasites can be explained by the reduced use of arginine in the H_N_ species as they primarily obtain energy from arginine catabolism [23, 33, 34]. This is consistent with previous studies of plant and animal proteins that observed reduced nitrogen content of amino acid side chains in the nitrogen-limited plant species [17, 35].

### Different metabolic strategies in the same host niche cause concomitant differences in gene sequence nitrogen content

To provide further insight into the relationship between metabolism and genome nucleotide composition, an additional analysis was conducted on Mollicutes parasites that occupy the same host niche but obtain energy through different metabolic strategies. Here, three Mollicutes species, *Mycoplasma hominis*, *Mycoplasma genitalium* and *Ureaplasma parvum* were analysed (note *Ureaplasma parvum* is also a Mollicute but a different species to *Mycoplasma parvum* used in the analyses above). Each of these three species reside in the same urogenital tract niche but obtain energy from catabolism of different biomolecules [23]. *M. genitalium* and *U. parvum* metabolise glucose and urea, respectively. However, *M. hominis* has lost the ability to generate ATP via glycolysis and instead generates ATP via nitrogen-liberating arginine catabolism [23].

**Fig. 3:**
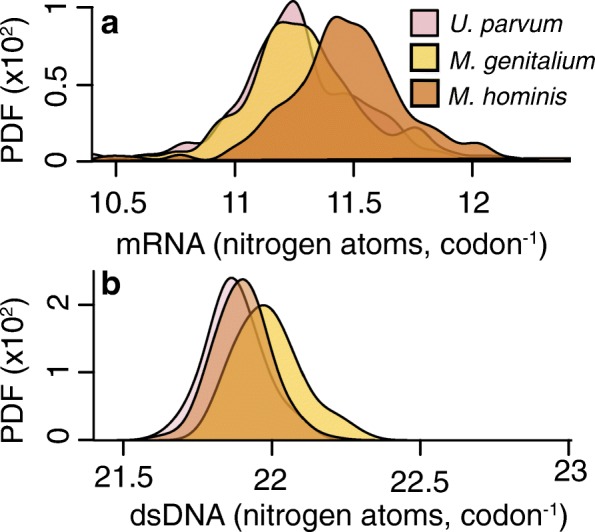
Analysis of gene nitrogen content of three parasites that occupy the same host niche but utilise different metabolic strategies. **a** The average mRNA nitrogen content per codon for 227 single copy genes present in each species. **b** The average nitrogen content per double-stranded codon (*dsDNA*) for the same genes. The *y-axis* is the probability density function (*PDF*) for the distributions. *Yellow* indicates an animal parasite that obtains energy from carbohydrate catabolism (medium nitrogen availability, *M*_*N*_). *Orange* indicates an animal parasite that obtains energy from amino acid and/or amino sugar catabolism (high nitrogen availability, *H*_*N*_). *Pink* indicates an animal parasite that obtains energy by exporting ammonia from the cell

Using the same methods outlined previously, 51,998 orthologous codons in 227 conserved single-copy orthologous genes (present in each of the three species) were compared (Fig. 3a). This revealed that despite inhabiting the same niche environment, there was a significant difference (*p <* 0.001) in the nitrogen cost of genes, equating to using one fewer nitrogen atom for every six codons in *M. hominis* (H_N_) compared to *M. genitalium* (M_N_) (~1.5 %). Since urea metabolism generates ammonia, one could expect *U. parvum* to be a H_N_ parasite. However, *U. parvum* exports ammonia to drive ATP synthesis, meaning energy generation is linked with export of nitrogen from the cell. Thus, analogous to *M. genitalium*, *U. parvum* is a nitrogen-limited species and uses one fewer nitrogen atom for every five codons compared to *M. hominis* (~1.8 %). As before, the strong mutation bias in Mollicutes means that the same nitrogen-dependent effect is not seen in their dsDNA (Fig. 3b). Taken together, this comparison reveals that, in a common host niche, different metabolic strategies can result in concomitant differences in mRNA nitrogen content.

### Differences in genome-wide patterns of synonymous codon use are explained by selection acting on codon nitrogen content

Given that there is a clear difference in the nitrogen content of genes between different nitrogen availability groups, it was assessed whether this phenomenon could be explained by differences in the nitrogen content of synonymous codons. To do this a novel model for genome-wide synonymous codon use was constructed that considers mutation bias and selection acting on the nitrogen content of codons (see the “A model for synonymous codon use under the joint pressures of selection and mutation bias” section in the “Methods”). Using this model, the value of the nitrogen-dependent selection bias (2*N*_*g*_ *s*) and mutation bias (*m*) were found that best explained the real sequence data (see “Methods” for complete model description). Here a negative value for 2*N*_*g*_ *s* indicates that selection is acting to decrease nitrogen content and vice versa.

**Fig. 4:**
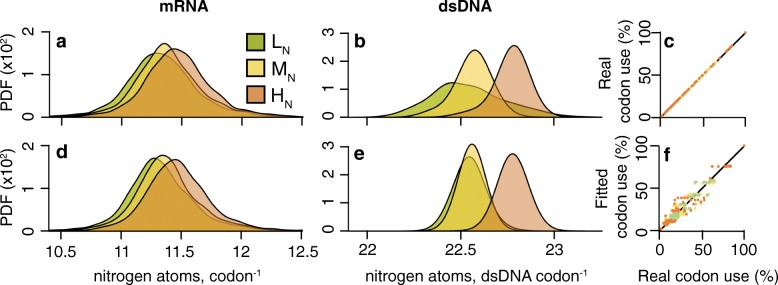
A selection-mutation model for synonymous codon use in kinetoplastids explains relative synonymous codon use with ~90 % accuracy and recapitulates the difference in the nitrogen cost of genes. **a** The average mRNA nitrogen content per codon for 3003 orthologous genes in the Kinetoplastida. **b** The average nitrogen content per double-stranded codon (*dsDNA*) for the genes in **a. c** Empirical codon use probabilities (expressed as percentages) plotted against themselves. **d** The average mRNA nitrogen content per codon for sequences simulated using synonymous codon use probabilities derived from fitting the selection-mutation model to the observed sequence data. **e** The average nitrogen content per double-stranded codon for the genes in **d. f** The synonymous codon use probabilities inferred using the selection-mutation model plotted against the empirical codon use probabilities expressed as percentages. *Dot colour* corresponds to nitrogen availability group. L_N_ R^2^ = 0.92, M_N_ R^2^ = 0.88, H_N_ R^2^ = 0.90

For the kinetoplastids, application of this modelling approach was able to explain genome-wide patterns of synonymous codon use with >90 % accuracy across all nitrogen availability groups (Fig. 4). Moreover, sequences simulated using these fitted codon use frequencies recapitulated the observed patterns of nitrogen content in mRNA (Fig. 4d) and dsDNA (Fig. 4e (Additional file 1: Figures S4 and S5). Consistent with nitrogen availability, the value of the selection bias for incorporation of nitrogen atoms in gene sequences was most negative in L_N_ parasites (2*N*_*g*_ *s* = −0.09), intermediate in M_N_ parasites (2*N*_*g*_ *s* = −0.06) and least negative in H_N_ parasites (2*N*_*g*_ *s* = −0.03). The distribution of 2*N*_*g*_ *s* parameters for individual species within each group were also significantly different between each group (ANOVA, *p* < 0.01). Thus, differences in nitrogen availability between species are reflected in the relative strengths of the selection bias on codon nitrogen content. Furthermore, mutation bias towards GC was lowest in L_N_ parasites (*m* = 0.67) and highest in H_N_ parasites (*m* = 0.31). Importantly, just considering selection acting on the nitrogen content of mRNA (Additional file 1: Figure S5b) or mutation bias (Additional file 1: Figure S5d) in isolation resulted in higher AIC values (Additional file 3: Table S1), indicating the dual parameter model is better. Thus, the pattern of codon use and gene nitrogen content is best explained by a model that considers both selection acting on the mRNA nitrogen content of genes and mutation bias (Fig. 4; Additional file 1: Figure S5e). Furthermore, the statistical significance of selection acting on the nitrogen content of coding sequences was assessed by a permutation test (see “Methods”). This showed that selection acting on the nitrogen content of the mRNA sequences was significant for L_N_ (*p* = 0.004) and M_N_ (*p* = 0.021) parasites but was not significant for the H_N_ kinetoplastid parasites (*p* = 0.457). This is consistent with our findings that indicate H_N_ kinetoplastids are not under selection to minimise the nitrogen content of their coding sequences. The change in codon bias also accounts for the majority of the difference in genome-wide GC content between species. Specifically, the coding regions constitute ~50 % of the genome in kinetoplastid parasites and thus changes in synonymous codon use account for 61 % of the observed difference in genome-wide GC content between H_N_ and L_N_ species (Additional file 3: Table S1).

It should be noted that simulating sequences using perfect genome-derived codon use frequencies (i.e. using 61 constrained parameters; Additional file 1: Figure S5h) results in simulated sequences whose distributions are not significantly different to those obtained in our two-parameter selection-mutation model. Thus, the difference between the distributions of nitrogen content for the real (Fig. 4b) and simulated sequences (Fig. 4e) is a result of factors affecting codon bias in individual genes that are not encapsulated by our genome-wide model.

**Fig. 5:**
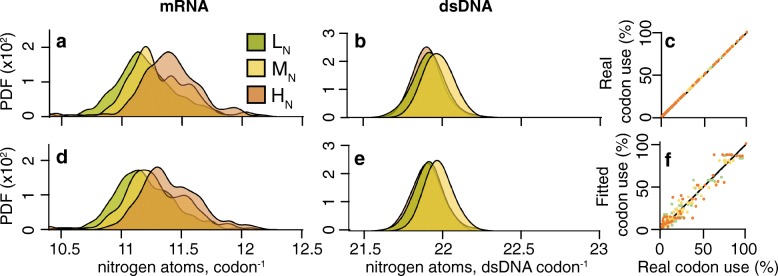
Model for synonymous codon use for Mollicutes explains relative synonymous codon use with ~94 % accuracy and recapitulates the difference in the nitrogen cost of genes. **a** The average mRNA nitrogen content per codon for 168 orthologous genes in the Mollicutes. **b** The average nitrogen content per double-stranded codon (dsDNA) for the genes in **a. c** Empirical codon use probabilities (expressed as percentages) plotted against themselves. **d** The average mRNA nitrogen content per codon for sequences simulated using synonymous codon use probabilities derived from fitting the selection-mutation model to the observed sequence data. **e** The average nitrogen content per double-stranded codon (dsDNA) for the genes in **d. f** The synonymous codon use probabilities inferred using the selection-mutation model plotted against the empirical codon use probabilities expressed as percentages. *Dot colour* corresponds to codon use probabilities for each nitrogen availability group. L_N_ R^2^ = 0.95, M_N_ R^2^ = 0.97, H_N_ R^2^ = 0.91

A similar phenomenon is observed for the Mollicutes, though the fitted mutation bias values are much larger (*m* > 3.5), indicative of a strong GC to AT mutation bias. This high value for *m* is consistent with the loss of dUTPase and a reduced ability to correct erroneous dUTP incorporation into the genome [31, 32]. The selection-mutation model is capable of explaining genome-wide patterns of codon use with 94 % accuracy across all nitrogen availability groups. Consistent with nitrogen availability, the value of the selection bias for incorporation of nitrogen atoms in gene sequences was most negative in L_N_ parasites (2*N*_*g*_ *s* = −0.24), intermediate in M_N_ parasites (2*N*_*g*_ *s* = −0.15) and least negative in H_N_ parasites (2*N*_*g*_ *s* = −0.13) (Fig. 5; Additional file 1: Figure S5m). The distribution of 2*N*_*g*_ *s* parameters for individual species within each group was significantly different when comparing L_N_ species with M_N_ or H_N_ (ANOVA, *p* < 0.01); however, the difference between M_N_ and H_N_ species failed to reach significance (ANOVA, *p* > 0.05) (Additional file 1: Figure S6). As for the kinetoplastids, the AIC values of the selection-mutation model were better than for the models that consider either selection or mutation bias individually (Additional file 1: Figure S5J, L; Additional file 3: Table S1). Furthermore, significance testing showed that selection acting on mRNA nitrogen content was significant for all Mollicutes groups (L_N_ *p* = 0.001, M_N_ *p* = 0.001, H_N_ *p* = 0.04). As coding sequences comprise the majority of these Mollicutes genomes (~83 %) the difference in genome-wide GC content between M_N_ and L_N_ species is fully attributable to differences in synonymous codon use (Additional file 3: Table S1).

To test whether the observed bias in codon use was also seen more broadly across the genome and not just in the conserved single copy genes, an additional analysis was conducted on all complete coding sequences (Additional file 3: Table S1). The pattern of codon bias was recapitulated for this larger gene set. However, the values obtained from the model when considering all complete coding sequences were less extreme than the values obtained when considering conserved orthologous sequences. This is expected as conserved sites in conserved genes have previously been shown to exhibit stronger codon bias [36].

### Gene expression negatively correlates with selection acting on mRNA nitrogen content

Selection acting on coding sequences is typically considered weak, especially given the low effective populations of the parasites in this study. However, previous studies have shown that selection is detectable in highly expressed genes [37–39] and most theories of codon usage predict that the degree of bias due to selection should increase with gene expression [40]. Given that there is a clear signature of selection acting on nitrogen content genome-wide, it was assessed whether the magnitude of this selection was a function of mRNA abundance. Here, the magnitude of selection acting on the nitrogen content of each gene was compared to the mRNA abundance of that gene. For each species there was a negative correlation between mRNA abundance and the fitted 2*N*_*g*_ *s* (Additional file 1: Figure S7). This shows that the strongest selection to minimise nitrogen content is observed in the most highly expressed genes. Moreover, the slope of the line was greatest for the L_N_ species, intermediate for the M_N_ species and weakest for the H_N_ species. This gene-level analysis is consistent with the genome-wide analysis that showed that L_N_ species have the greatest selective pressure to minimise nitrogen use.

### Low nitrogen availability (L_N_) parasites have ribosomal RNA sequences that use the lowest amount of nitrogen

Ribosomal RNA (rRNA) typically constitutes the majority of RNA within a cell. To investigate whether selection acting on nitrogen content extends beyond coding sequences, the total nitrogen content of rRNA per ribosome was calculated. Consistent with the analysis of coding sequences, L_N_ parasite rRNAs require the lowest amount of nitrogen. In the Mollicutes, L_N_ parasites used eight fewer nitrogen atoms compared to M_N_ and 63 fewer atoms compared to H_N_ parasites per 70S ribosome (Additional file 3: Table S1). This difference is lower than expected when compared to the analysis of protein-coding genes. Given the length of the rRNA sequence analysed, a difference of 77 and 140 nitrogen atoms would have been predicted. This reduced difference is most likely due to structural constraints on rRNA and the fact that it is not composed of codons and so may lack the flexibility provided by synonymous codons.

The same analysis of rRNA sequences was carried out for the kinetoplastids. Consistent with the analysis of the Mollicutes, the RNA component of the 80S ribosome required the least amount of nitrogen in the L_N_ kinetoplastid parasites. However, due to large insertions in *Trypanosoma cruzi* rRNAs, the M_N_ parasites required more nitrogen than the H_N_. These inserted regions increased the total nitrogen content in the *T. cruzi* rRNA by >1500 nitrogen atoms (~7 % more than the other M_N_ species; Additional file 3: Table S1). Thus, with one exception, the analysis of rRNA genes is consistent with the analysis of protein-coding genes.

### Nitrogen content of nucleotide sequences can predict metabolic capability

**Fig. 6:**
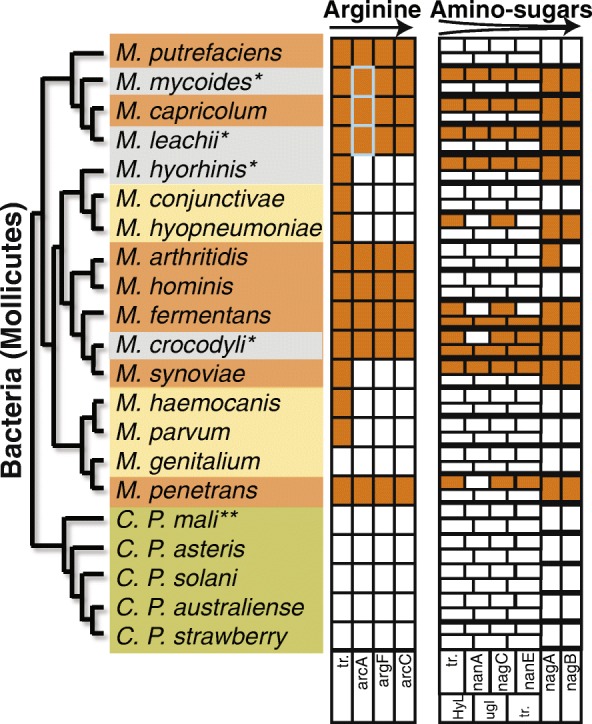
Selection-mutation model of codon use can predict the metabolic capacity of parasites from raw nucleotide sequences. L_N_ (*green*), M_N_ (*yellow*) and H_N_ (*orange*) are species with low, medium and high nitrogen availability, respectively. Species denoted with an *asterisk* (*grey*) are the new species, each of which is predicted to be a H_N_ parasite. The presence of named genes involved in arginine catabolism and amino sugar catabolism is indicated by *orange boxes*. Gene names are provided below the boxes and the abbreviations correspond to the following genes: *tr.* transporter, *arcA* arginine deiminase, *argF* ornithine carbamoyltransferase, *arcC* carbamate kinase, *nanA* N-acetylneuraminate lyase, *nanK/nagC* N-acetylmannosamine kinase, *nanE* N-acetylmannosamine-6-p epimerase, *nagA* N-acetyl-D-glucosamine-6-phosphate amidohydrolase, *nagB* D-glucosamine-6-phosphate aminohydrolase, *HyL* hylauronate lyase, *ugl* glucuronidase. *Grey outlined boxes* encode *aguA*, whose protein product catalyses the same reaction as the product of *arcA* on an equivalent substrate

Given that the relative use of synonymous codons is affected by selection acting on nitrogen content, it was determined to what extent the selection-mutation model could predict the dietary nitrogen content of an organism. This was tested by analysing four additional Mollicute genomes not included in the original analysis. Each additional species was classified as H_N_ by model selection through maximum likelihood estimation (Additional file 3: Table S1). To provide support for these classifications, the parasites’ genomes were searched for genes required for amino acid and amino sugar catabolism. This revealed that, in contrast to M_N_ Mollicutes parasites, the genomes of the additional species each encoded complete metabolic pathways for catabolism of either arginine and/or amino sugars (Fig. 6; Additional file 3: Table S1). Moreover, the genes for these pathways were co-located in gene clusters, indicative of genes belonging to the same metabolic pathway (Additional file 1: Figure S8). These results demonstrate the utility of the model for providing information about the metabolic capabilities of an organism from raw nucleotide sequences.

### Selection acting on nitrogen content is independent of selection acting on translational efficiency

Translational selection, which is a function of the number of iso-accepting tRNAs encoded in a genome, has long been considered a major driver of codon bias [8]. To determine how selection acting on nitrogen content acts in concert with selection acting on translational efficiency (tAI), the model was expanded to include tAI as an additional parameter (see “Methods”). For the Mollicutes, unlike the result above where selection acting on nitrogen content was significant for all three parasite groups, it was found that considering tAI values alone or in conjunction with mutation bias was not significantly better than when tAI was omitted (*p* > 0.05). However, when all three parameters (nitrogen content, mutation bias and tAI) were considered together, the model fits the data significantly better than when considering just selection acting on nitrogen content and mutation bias for the L_N_ and H_N_ parasites (*p* ≤ 0.02). Thus, selection acting on translational efficiency is independent of selection acting on nitrogen content and only provides a significant contribution to codon bias in L_N_ and H_N_ species (Additional file 1: Figure S5).

In contrast, for the kinetoplastids it was found that the fit to observed patterns of codon use was significantly better with the inclusion of tAI values in conjunction with mutation bias (L_N_ *p* = 0.018, M_N_ and H_N_ *p* = 0). The contribution of tAI was also significant for all three kinetoplastid parasite groups when all three parameters (nitrogen content, mutation bias and tAI) were considered together (L_N_ *p* = 0.006, M_N_ *p* = 0.001, H_N_ *p* = 0; Additional file 1: Figure S5). Thus, as for the Mollicutes, selection acting on translational efficiency is independent of selection acting on nitrogen content. Furthermore, inclusion of translational efficiency in the model improves overall fit by ~2 % to give an average accuracy of 94.3 %. This compares to a 3.1 % improvement in overall fit when selection acting on nitrogen content is added to the model that only considers mutation bias and translational efficiency.

Selection acting on nitrogen content can explain why the most translationally optimal codons are not always the codons that are most frequently used. For example, in Mollicutes parasites only 33 % of the most frequently used codons for each amino acid are the most translationally efficient while 66 % are those with the lowest nitrogen content (Additional file 1: Figure S9). A similar pattern occurs in the kinetoplastid parasites, although the most translationally efficient codon is the most frequently used codon more often than the most nitrogen-efficient codon (74 % compared to 30 %, respectively). This interplay between translation and nitrogen content is also seen when the relative order of all synonymous codons is analysed in these two parasite groups (Additional file 1: Figure S9). Furthermore, these observations are consistent with the global analysis of codon use presented above which showed that selection acting on nitrogen content was more important than selection acting on translation efficiency in determining patterns of codon bias in Mollicutes, while selection acting on nitrogen content and translation efficiency was required to explain patterns of codon use in kinetoplastids.

## Discussion

Studies on the interactions between diet, metabolism and evolution have primarily focused on the presence or absence of individual genes in the context of specific metabolic pathways. However, the impact of an organism’s diet on the evolution of its genes and genome is poorly understood. Here we show that differential nitrogen availability, due to differences in host environment and metabolic inputs, alters synonymous codon usage and thus gene sequence evolution in both bacterial and eukaryotic parasites. Moreover, this impact is sufficient to enable prediction of metabolic inputs of parasites from comparative analysis of the nucleotide composition of orthologous genes.

In this work we provide a novel selection-mutation model for synonymous codon use that builds upon a strong theoretical foundation [41–43]. In this model we have amalgamated multiple factors contributing to genome-wide GC content into the single variable termed mutation bias (*m*, mutation bias towards AT). Such factors include the bias of an organism’s DNA polymerase [44], gene conversion [45], differences in repair efficiency [32] as well as mutational biases during DNA replication [1–3]. We also suggest that differences in nitrogen availability may also contribute to differences in mutation bias through influencing the relative abundance of nucleotides [20]. Considering mutation bias alone was able to recapitulate the observed synonymous codon use with ~90 % accuracy for both the Mollicutes and kinetoplastid parasites. Furthermore, the large differences in mutation bias between kinetoplastids (*m* < 1) and Mollicutes (*m* > 3.5) is able to explain the large differences in observed patterns of codon bias between the two distantly related parasite lineages. Interestingly, the kinetoplastid *m* values are each below 1 (L_N_ *m* = 0.68, M_N_ *m* = 0.74, H_N_ *m* = 0.31) and thus correspond to a bias towards GC. The differences in mutation bias between parasite groups is consistent with differences in nitrogen availability, as a high GC content is equivalent to high nitrogen content of the dsDNA. An analogous nitrogen-dependent difference in *m* is not seen in the Mollicutes. We propose that this is due to the strong AT mutation bias (*m* values all greater than 3.5) that constrains dsDNA nitrogen within a narrow range of values compared to the kinetoplastids.

Due to the complementary nature of DNA, a change on either DNA strand will cause a corresponding change on the other strand. Therefore, mutation bias alone was unable to produce the differences in the nitrogen content of the coding strand (i.e. the mRNA) that was observed between species with different nitrogen availabilities. As shown in Eq. 2, selection depends on N_g_, the effective number of genes at the locus in the population, which is linked to the effective population size (N_e_) of an organism [46]. Organisms with low long-term effective population sizes have a reduced impact from selection due to the greater impact of random genetic drift. Thus, N_g_ plays an important role in determining the role of selection in biased codon usage. As has been noted before, however, evaluating the long-term N_g_ value for an organism is very difficult [47]. Eukaryotes have lower N_g_ values than prokaryotes and parasites in general have lower N_g_ values than their free-living counterparts due to clonal life stages and bottlenecks during transmission. Our model evaluates the selection bias acting on nitrogen content using a composite parameter (2*N*_*g*_ *s*). Thus, the value of the selection coefficient *s* is linearly dependent on estimates of N_g_ (i.e. increasing N_g_ by a factor of 10 decreases *s* by a factor of 10). It is interesting to note that estimates of N_g_ for prokaryotes and unicellular eukaryotes differ by a factor of 10 [46], similar to the magnitude of difference we see between 2*N*_*g*_ *s* for the Mollicutes and the kinetoplastids, indicating that the selection coefficient *s* may be similar for the two distantly related groups.

Previous studies investigating the role of selection in codon bias have revealed that selection acting on translational efficiency in Mollicutes is marginal [47]. Although codon biases in prokaryotic genomes are associated with gene expression levels [48, 49], in some cases the optimal codons disagree with the tRNA composition. These observations support the results presented here which show that, for Mollicutes, inclusion of tAI values does not significantly improve the fit of the model unless it is considered in conjunction with both mutation bias and selection acting on the nitrogen content of coding sequences. Our finding that selection acts on the nitrogen content of codons provides a novel mechanism that links codon usage bias to metabolism and environment. Furthermore, as the model developed here is sufficient to enable prediction of metabolic inputs from gene sequences, it may have application in interrogating metagenome data and genome data from shotgun sequencing of microbial communities where metabolic requirements are unknown.

Though the selection-mutation model provides considerable explanatory power for the species used in this analysis, it does not perfectly re-capitulate the observed patterns of codon use. This is most likely due to the fact that specific sites within a gene will be under different pressures that cannot be captured by a genome-wide approach. For example, factors indirectly related to protein translation, such as mRNA secondary structures at the 5′ region of a gene, have been shown to be under selection for efficient binding of ribosomes to mRNAs and hence can have a weak effect on the frequency of codon usage at those sites [50]. A more complex model could include variation in codon bias between genes due to gene-specific selective pressures such as splice site conservation, mRNA stability, ribosome binding and mRNA abundance. Taken together these factors may account for the ~6 % of missing variation not explained by the selection-mutation model presented here. Incorporation of these factors into the model would be an interesting avenue of future research.

Thermophilic bacteria purine-load their genomic sequences to the extent that amino acid composition is affected [10]. However, this effect is not seen in the mRNA of mesophilic organisms [51, 52] and so would not be expected to feature in the dataset analysed here. For example, the difference observed between M_N_ and H_N_ parasites cannot be due to temperature as both groups infect animal hosts with very similar (if not identical) temperatures. Furthermore, some of the H_N_ (*Mycoplasma crocodyli* and *Leishmania tarentolae*) and M_N_ (*Trypanosoma grayi*) species in both the Mollicutes and the kinetoplastids infect cold-blooded reptiles and thus would have host temperatures more similar to plant-infecting L_N_ parasites than to warm blooded animals. Even though these parasites infect cold-blooded animals, their nitrogen use profiles are consistent with their metabolic group rather than their host temperature. Finally, conducting our analysis on parasites in the same ecological niche revealed that, at the same temperature, in the same microenvironment, the parasites exhibited different nucleotide nitrogen content consistent with their dietary nitrogen availability. These results indicate that while temperature may be important in extreme environments, temperature is not a factor in the comparisons presented here. This is consistent with previous analyses that showed that even at relatively freely evolving sites, mRNA GC content did not appear to be adapted to the thermal environment [52].

## Conclusions

This analysis demonstrates via multiple complementary approaches that differential nitrogen availability, due to differences in host environment and metabolic inputs, contributes to changes in codon bias and genome composition. Specifically, adaptation to low nitrogen availability results in reduced nitrogen content in nucleotide sequences. These results reveal a previously hidden relationship between cellular metabolism and genome evolution and provide new insight into how genome sequence evolution can be influenced by adaptation to different diets.

## Methods

### Data sources

We obtained 17 Mollicutes genomes from the NCBI GenBank. These comprised four plant glycolytic parasite species (*Candidatus Phytoplasma asteris* [53], *Candidatus Phytoplasma austrailense* [54], *Candidatus Phytoplasma mali* [55], *Candidatus Phytoplasma solani* [56], *Candidatus Phytoplasma strawberry* [57]), five animal glycolytic parasite species (*Mycoplasma conjunctivae* [58], *Mycoplasma genitalium* [59], *Mycoplasma haemocanis* [60], *Mycoplasma hyopneumoniae* [61], *Mycoplasma parvum* [62]) and seven parasite species known to obtain energy from catabolism of amino acids or amino sugars (*Mycoplasma arthritidis* [63], *Mycoplasma capricolum* [PRJNA16208], *Mycoplasma fermentans* [64], *Mycoplasma hominis* [23], *Mycoplasma penetrans* [65], *Mycoplasma putrefaciens* [66], *Mycoplasma synoviae* [67]). A further four parasite species were used for testing the predictive capacity of the model for synonymous codon use (*Mycoplasma crocodyli* [68], *Mycoplasma hyorhinis* [69], *Mycoplasma leachii* [70], *Mycoplasma mycoides* [70]).

15 kinetoplastid genomes were obtained online from TriTrypDB [71], NCBI genbank or the European Nucleotide Archive. These comprised four plant glycolytic parasite species (*Phytomonas EM1* [GCA_000582765] [72], *Phytomonas françai* [PRJNA343003], *Phytomonas HART1* [GCA_000982615] [72], *Phytomonas serpens* [PRJNA80957], 5 animal glycolytic parasite species (*Trypanosoma brucei* [PRJNA15565], *Trypanosoma congolense* [PRJNA12958], *Trypanosoma cruzi* [PRJNA15540/PRJNA11755], *Trypanosoma grayi* [PRJNA258390], *Trypanosoma vivax* [PRJNA12957]) and six parasite species who obtain energy primarily from catabolism of amino acids (*Leishmania braziliensis* [PRJNA19185], *Leishmania donovani* [PRJNA171503], *Leishmania infantum* [PRJNA19187], *Leishmania major* [PRJNA10724], *Leishmania* *mexicana* [PRJNA172192], *Leishmania tarentolae* [PRJNA15734]).

### Inference of orthogroups and construction of multiple sequence alignments

The predicted amino acid sequences for each species were subject to orthogroup inference using OrthoFinder [73] using the default program parameters. Single copy genes were selected for analysis to ensure orthology and so that paired comparisons could be made; i.e. a single-copy orthologous gene that is present in two different species can be treated as a paired observation. Single copy gene orthogroups were further filtered to retain those that had representation from at least three species per group (L_N_, M_N_ and H_N_). Protein sequences for these orthogroups were aligned using MergeAlign [74]. The corresponding coding sequences were re-threaded back through the aligned amino acid sequences using custom Perl scripts. These multiple sequence alignments were then filtered so that only un-gapped columns that obtained a MergeAlign column score of >0.75 were retained for further analysis. These stringent filtration criteria ensured that only high accuracy, unambiguously aligned orthologous positions were used for all analyses. The accession numbers for the full set of orthogroups used in this analysis are provided in Additional file 3: Table S1.

### Evaluation of nitrogen content of nucleotide sequences

The filtered multiple sequence alignments above were used to calculate the number of nitrogen atoms used per codon, per gene per species. The number of nitrogen atoms per codon per gene was evaluated as the arithmetic mean of the number of nitrogen atoms in the filtered aligned codons for that gene described above. The average number of nitrogen atoms contained within the mRNA and the dsDNA were recorded for each gene. These data were plotted as probability density functions using the R density distribution plot function with the total area under each curve equal to one.

### Analysis of rRNA

A database of representative rRNA sequences was generated and blasted against the genomes of all the parasites in this study to find the locations of the rRNAs. In the event of no or partial blast hits, sequences were downloaded from NCBI and the accession numbers noted in Additional file 3: Table S1. Sequences were then aligned using MAFFT [75] to identify the true start and end of the rRNA molecules. The nitrogen content of these sequences was calculated. Due to difficulties in sequencing and assembling repetitive rDNA loci, some species did not have complete sequences to include in this analysis. Those were labelled NF (not found).

### Statistical tests

Given that single copy orthologous genes present in different species can be treated as paired observations, Wilcoxon signed-rank tests were used to compare nitrogen content between different parasite groups. In each case the null hypothesis was that the difference between the two groups was due to chance (symmetric around zero). The alternative hypothesis was that the difference in nitrogen content between each group was not due to chance. In all cases, the test used was two-tailed so that either a greater or lesser nitrogen content difference would reject the null hypothesis. Pairing of samples is justified as the paired observations (genes) are orthologous and descended from the same common ancestor under different environmental and metabolic conditions.

Goodness of fit and the statistical significance of the inclusion of additional parameters to the model were assessed by comparison of AIC values and by using a permutation test, respectively. For the permutation test, the log likelihood values obtained by the model when run with real values were compared with the log likelihood values obtained by the model when it was run with shuffled/randomised values. To analyse the significance of the inclusion of nitrogen selection to the model, the codon nitrogen contents were calculated and then shuffled to randomly assign the values to each codon. The model was then fit to the data using these randomised values and the log likelihood compared to the log-likelihood obtained using the real values. This was repeated for 1000 independently shuffled sets. The same principle was applied to significance testing of the tAI values. An example of the distributions generated when codon nitrogen content was shuffled is provided in Additional file 1: Figure S10.

### A model for synonymous codon use under the joint pressures of selection and mutation bias

To determine whether nitrogen availability influences interspecies variation in codon use and nucleotide content, a model for synonymous codon use was constructed. This model considers the selection bias acting to modulate a codon’s nitrogen content and an organism’s mutation bias. The system of equations describing the model are as follows.

#### Synonymous codon use considering selection acting on mRNA nitrogen content

Here we consider that selection acts to bias synonymous codon use in proportion to the number of nitrogen atoms contained within each codon, i.e.:

1 $$ S\left({\mathcal{C}}_i\right)=s{N}_{mRNA} $$

where $S\left({\mathcal{C}}_i\right)$ is a measure of the relative fitness of codon ${\mathcal{C}}_i$, with *N*_*mRNA*_ being the number of nitrogen atoms in codon ${\mathcal{C}}_i$ and *s* being the selection coefficient. Following previous published work [41–43], we model the selection bias towards codon ${\mathcal{C}}_i$ as:

2 $$ \alpha \left({\mathcal{C}}_i\right)={e}^{2{N}_gS\left({\mathcal{C}}_i\right)} $$

where $\alpha \left({\mathcal{C}}_i\right)$ is the selection bias towards codon ${\mathcal{C}}_i$ and *N*_*g*_ is the effective number of genes at a locus. Only considering this selection bias, we evaluate the genome-wide probability of observing codon ${\mathcal{C}}_i$ for amino acid θ as:

3 $$ p\left({\mathcal{C}}_i\ \Big|\ \theta \right) = \frac{\alpha \left({\mathcal{C}}_i\right)}{{\displaystyle {\sum}_{\theta }}\alpha \left(\mathcal{C}\right)} $$

That is, the probability of observing codon ${\mathcal{C}}_i$ is the selection bias towards codon ${\mathcal{C}}_i$ divided by the sum of selection biases for all codons encoding amino acid θ. Equation 3 satisfies the law of total probability such that the sum of the probabilities of observing of all the codons that encode the same amino acid sum to one.

#### Synonymous codon use considering mutation bias only

Mutation bias is known to be influenced by a range of factors including but not limited to the bias of an organism’s polymerase-α subunit [44], gene conversion [45] and differences in repair efficiency [32]. We propose that nitrogen-mediated changes in nucleotide pools also contribute to this mutation bias, as changes in nucleotide pools result in changes in mutation bias [20]. For example, the amount of biologically available nitrogen within a cell could alter the relative abundance of nucleotides via enzymes such as CTP synthase that catalyse nitrogen-dependent nucleotide interconversion of UTP and CTP. Here we have amalgamated these factors into the single variable *m*.

4 $$ \delta = \frac{m}{m+1} $$

where *δ* is the probability that a particular site is A or T given a mutation bias towards AT of *m* as previously described [46]. Due to base pairing, the probability of A or T is equivalent. This equation assumes that the nucleotide composition of the genome is at equilibrium and that the mutation rate per site is independent of the status of neighbouring sites [46]. For example, if there is no mutation bias towards AT or GC, *m* will be 1 and *δ* will be 0.5 and thus there is an equal likelihood of any site being AT or GC. We model the mutation bias towards codon ${\mathcal{C}}_i$ as:

5 $$ \beta \left({\mathcal{C}}_i\right)={\delta}^{AT}{\left(1-\delta \right)}^{GC} $$

where $\beta \left({\mathcal{C}}_i\right)$ is the mutation bias towards codon ${\mathcal{C}}_i$, *AT* is the number of A or T nucleotides in codon ${\mathcal{C}}_i$ and *GC* is the number of G or C nucleotides in codon ${\mathcal{C}}_i$. Considering only mutation bias we evaluate the genome-wide probability of observing codon ${\mathcal{C}}_i$ for amino acid θ as:

6 $$ p\left({\mathcal{C}}_i\ \Big|\ \theta \right) = \frac{\beta \left({\mathcal{C}}_i\right)}{{\displaystyle {\sum}_{\theta }}\beta \left(\mathcal{C}\right)} $$

That is, the probability of observing codon ${\mathcal{C}}_i$ is the mutation bias towards codon ${\mathcal{C}}_i$ divided by the sum of mutation biases for all codons encoding amino acid θ. Equation 6 also satisfies the law of total probability such that the sum of the probabilities of observing all the codons that encode the same amino acid sum to one. For example, if *m* = 3 then *δ* = 0.75 and we consider amino acid C (encoded by codons TGC and TGT), then the mutation bias towards codon TGC = *β*(*TGC*) = 0.75^1^(1 − 0.75)^2^ = 0.047 and the mutation bias towards codon TGT = *β*(*TGT*) = 0.75^2^(1 − 0.75)^1^ = 0.141. Thus, the genome-wide probability of observing codon $\mathrm{T}\mathrm{G}\mathrm{C} = \kern0.5em \frac{0.047}{0.047+0.141}\kern0.75em  = 0.25$ and the genome-wide probability of observing codon TGT = 0.75.

#### A model for synonymous codon use under the joint pressures of selection and mutation bias

We model the bias towards codon ${\mathcal{C}}_i$ under the joint pressures of selection and mutation as the product of Eqs. 2 and 5.

7 $$ \gamma \left({\mathcal{C}}_i\right)=\alpha \left({\mathcal{C}}_i\right)\beta \left({\mathcal{C}}_i\right) $$

As above we evaluate the genome-wide probability of observing codon ${\mathcal{C}}_i$ for amino acid θ as:

8 $$ p\left({\mathcal{C}}_i\ \Big|\ \theta \right)=\frac{\gamma \left({\mathcal{C}}_i\right)}{{\displaystyle {\sum}_{\theta }}\gamma \left(\mathcal{C}\right)} $$

It should be noted that selection in this model only considers the nitrogen content of a codon and does not consider other factors such as biased gene conversion [46]. However, kinetoplastids primarily reproduce by clonal expansion and the prokaryotic genomes are haploid; thus, gene conversion may have limited impact in these organisms.

#### Calculation of codon tRNA adaptation index values

The tRNA adaptation index (tAI) [76] of a codon takes into account both the abundance of iso-accepting tRNAs and wobble-base pairing to evaluate the efficiency of translation of a given codon. Using the equation developed by dos Reis et al. [77] below and the optimised *s*_*ij*_ values obtained by Tuller et al. [78], tAI values for each codon were evaluated:

9 $$ \omega \left({C}_i\right)=\kern0.75em {\sum}_{j=1}^{n_i}\kern0.5em \left(\ 1 - {s}_{ij}\right)tGC{N}_{ij} $$

where *ω*(*C*_*i*_) is the absolute adaptiveness value for each codon *C*_*i*_ (referred to in the rest of the text as the tAI value), *n*_*i*_ is the number of tRNA isoacceptors that recognise codon *C*_*i*_, *tGCN*_*ij*_ is the gene copy number of the *j* ^th^ tRNA that recognises codon *C*_*i*_, and *s*_*ij*_ is the selective constraint on the efficiency of codon-anticodon coupling.

We model the translational selection bias towards codon ${\mathcal{C}}_i$ as:

10 $$ \upeta \left({\mathcal{C}}_i\right)={e}^{2{N}_g\sigma \omega \left({\mathcal{C}}_i\right)} $$

where $\omega \left({\mathcal{C}}_i\right)$ is the translational selection bias towards codon ${\mathcal{C}}_i,\kern0.5em \sigma$ is the selection coefficient and *N*_*g*_ is the effective number of genes at a locus.

As above we evaluate the genome-wide probability of observing codon ${\mathcal{C}}_i$ for amino acid θ as:

11 $$ p\left({\mathcal{C}}_i\ \Big|\ \theta \right) = \frac{\eta \left({\mathcal{C}}_i\right)}{{\displaystyle {\sum}_{\theta }}\eta \left(\mathcal{C}\right)} $$

When considering all three parameters (mutation bias, selection acting on the nitrogen content of coding sequences and translational selection) we model the bias towards codon ${\mathcal{C}}_i$ as the product of Eqs. 2, 5 and 10.

12 $$ \varepsilon \left({\mathcal{C}}_i\right)=\alpha \left({\mathcal{C}}_i\right)\beta \left({\mathcal{C}}_i\right)\upeta \left({\mathcal{C}}_i\right) $$

As above we evaluate the genome-wide probability of observing codon ${\mathcal{C}}_i$ for amino acid θ as:

13 $$ p\left({\mathcal{C}}_i\ \Big|\ \theta \right) = \frac{\varepsilon \left({\mathcal{C}}_i\right)}{{\displaystyle {\sum}_{\theta }}\varepsilon \left(\mathcal{C}\right)} $$

### Model fitting and implementation

Using the system of equations in the model, the parameters (2*N*_*g*_ *s*, *m *and tAI) were estimated for each of the parasite groups using a maximum likelihood approach. The models for both the Mollicute and kinetoplastid parasites each contain a maximum of three free parameters (selection acting on nitrogen content, mutation bias and translational efficiency) and thus a brute-force parameter search was conducted to find their optimal values. Here, the likelihood of observing the set of sequences contained within each parasite group was evaluated given the model for synonymous codon use and the values of the parameters. It was evaluated as follows:

$$ \mathrm{\mathcal{L}}\left(s,m\Big|X\right) = {\displaystyle \prod_{{\mathcal{C}}_i}}p{\left({\mathcal{C}}_i\Big|\theta \right)}^{N_{{\mathcal{C}}_i}} $$

where *X* is the set of coding sequences for a given species and ${N}_{{\mathcal{C}}_i}$ is the number of times that codon ${\mathcal{C}}_i$ occurs in the set of sequences *X.* The optimal parameter values were determined as those with the maximum likelihood. This was applied to look at both orthologous genes (the same set as those described in the “Inference of orthogroups and construction of multiple sequence alignments” section) and the full set of coding sequences. Source code and data files for this analysis are available from the Zenodo research data repository (https://doi.org/10.5281/zenodo.154493).

### Classification of additional species using the metabolic model for synonymous codon use

Four additional Mollicutes genomes not included in the initial analysis were downloaded from NCBI to test the ability of the model for synonymous codon use to predict the metabolic properties of these organisms from analysis of codon use. These species were *M. crocodyli*, *Mycoplasma hyorhinis*, *Mycoplasma leachii* and *Mycoplasma mycoides.* Based on literature evidence and phylogeny (Fig. 6), it was expected that some of the additional species would be classified as H_N_ and some as M_N_ parasites. Using the system of equations described above and the values obtained for the dependency parameters (2*N*_*g*_ *s* and *m*) for each of the L_N_, M_N_ and H_N_ Mollicutes parasite groups, a likelihood that each species belonged to each group was calculated (Additional file 3: Table S1). The model with the highest likelihood was determined to be H_N_ in all instances. This classification was confirmed using a Wilcoxon signed-rank test on the nitrogen cost of the mRNA. Each of the additional species was significantly different (*p* < 0.001) from the L_N_ and M_N_ groups and not significantly different (*p* > 0.05) from the H_N_ group. The one exception to this was *M. crocodyli.* This species had the highest mRNA nitrogen cost of any Mollicutes species in this analysis and was significantly higher than the other species in the H_N_ group. This may indicate increased dependence on nitrogen liberating metabolic pathways or an increased availability of nitrogen in the host environment.

## Additional files

Additional file 1: Supplemental figures. (PDF 3133 kb)
Additional file 2: Supplemental information on parasite metabolism. (PDF 425 kb)
Additional file 3: Sheet 1: Accession numbers and corresponding orthogroups for all kinetoplastid species used in this analysis. Sheet 2: Accession numbers for the genes required for arginine and amino sugar metabolism in the Mollicutes. Sheet 3: The model was run on the dataset of orthologous genes (as described in the “Inference of orthogroups and construction of multiple sequence alignments” section in the “Methods”) and it was also run separately on all complete coding sequences. A complete coding sequence was considered as any coding sequence with start and stop codons whose length was divisible by 3 and longer than 30 nucleotides. This sheet shows the parameters obtained for each of the L_N_, M_N_ and H_N_ groups for both the Mollicutes and kinetoplastid parasites. Highlighted in *yellow* are the parameters obtained for the original two-parameter model that only considers selection acting on nitrogen content in coding sequences and mutation bias. Highlighted in *green* are the values obtained for the three-parameter model, which also considers selection acting on translational efficiency (tAI). The model parameters that produce the highest log likelihood values and the lowest AIC values are the best fit to the observed data. Sheet 4: Genome locations, nitrogen use and aligned sequences for Mollicutes 5S, 16S and 23S rRNA. Total nitrogen used in rRNA per 70S ribosome is given in the top left corner. Sheet 5: Genome locations, nitrogen use and aligned sequences for kinetoplastid 5.8S, 18S, 23S alpha and 23S beta rRNA. Total nitrogen used in rRNA per 80S ribosome is given in the top left corner. Sheet 6: Accession numbers for the genes required for arginine and amino sugar metabolism in the Mollicutes. (XLSX 639 kb)
