# Supplementary material for: Dietary nitrogen alters codon bias and genome composition in parasitic microorganisms
Source: Genome Biol. 2016 Nov 15;17:226. doi: 10.1186/s13059-016-1087-9 (PMC5109750; doi:10.1186/s13059-016-1087-9)
Supplement: Additional file 1: — Supplemental figures. (PDF 3133 kb) [file 13059_2016_1087_MOESM1_ESM.pdf]

Supplemental Fig. S1.

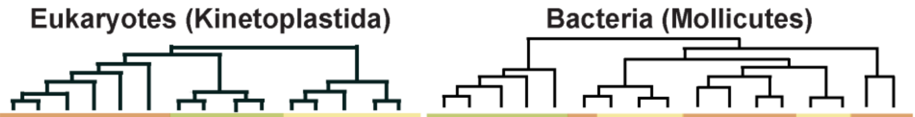

<table>
<tr><th></th><th>Genome size (Mb)</th><th>% GC</th><th>Host 1</th><th>Host 2 (vector)</th><th>Energy source in Host 1</th><th>Energy source in Host 2</th></tr>
<tr><td>M. putrefaciens</td><td>0.8</td><td>27</td><td>Goat</td><td>None</td><td>G | AA</td><td></td></tr>
<tr><td>M. capricolum</td><td>1.0</td><td>24</td><td>Ruminant</td><td>None</td><td>G | AA | AS</td><td></td></tr>
<tr><td>M. conjunctivae</td><td>0.9</td><td>29</td><td>Sheep/Goat</td><td>None</td><td>G</td><td></td></tr>
<tr><td>M. hyopneumoniae</td><td>0.9</td><td>29</td><td>Swine</td><td>None</td><td>G</td><td></td></tr>
<tr><td>M. arthritidis</td><td>0.8</td><td>31</td><td>Rats/Mice</td><td>None</td><td>AA</td><td></td></tr>
<tr><td>M. hominis</td><td>0.7</td><td>27</td><td>Human</td><td>None</td><td>AA</td><td></td></tr>
<tr><td>M. fermentans</td><td>1.1</td><td>27</td><td>Human</td><td>None</td><td>G | AA | AS</td><td></td></tr>
<tr><td>M. synoviae</td><td>0.8</td><td>28</td><td>Bird</td><td>None</td><td>G | AS</td><td></td></tr>
<tr><td>M. haemocanis</td><td>0.9</td><td>35</td><td>Dog</td><td>None</td><td>G</td><td></td></tr>
<tr><td>M. parvum</td><td>0.6</td><td>27</td><td>Swine</td><td>None</td><td>G</td><td></td></tr>
<tr><td>M. genitalium</td><td>0.6</td><td>31</td><td>Human</td><td>None</td><td>G</td><td></td></tr>
<tr><td>M. penetrans</td><td>1.4</td><td>26</td><td>Human</td><td>None</td><td>G | AA</td><td></td></tr>
<tr><td>C. P. mali</td><td>0.6</td><td>21</td><td>Apple</td><td>Cacopsylla melanoneura</td><td>Malate catabolism</td><td>Malate catabolism</td></tr>
<tr><td>C. P. asteris</td><td>0.9</td><td>26</td><td>Onion</td><td>Multiple leafhoppers</td><td>G</td><td>G</td></tr>
<tr><td>C. P. solani</td><td>0.6</td><td>29</td><td>Tomato</td><td>Hyalesthes obsoletus</td><td>G</td><td>G</td></tr>
<tr><td>C. P. australiense</td><td>0.9</td><td>27</td><td>Strawberry</td><td>Oliarus atkinsoni</td><td>G</td><td>G</td></tr>
<tr><td>C. P. strawberry</td><td>1.0</td><td>27</td><td>Strawberry</td><td>Multiple leafhoppers</td><td>G</td><td>G</td></tr>
<tr><td>T. grayi</td><td>21.0</td><td>54</td><td>Crocodile</td><td>Glossina sp.</td><td>G</td><td>G | AA</td></tr>
<tr><td>T. cruzi</td><td>32.5</td><td>51</td><td>Mammals</td><td>Triatominae</td><td>G</td><td>G | AA</td></tr>
<tr><td>T. vivax</td><td>47.5</td><td>54</td><td>Mammals</td><td>Glossina sp.</td><td>G</td><td>G | AA</td></tr>
<tr><td>T. congolense</td><td>41.4</td><td>48</td><td>Mammals</td><td>Glossina sp.</td><td>G</td><td>G | AA</td></tr>
<tr><td>T. brucei</td><td>35.8</td><td>47</td><td>Mammals</td><td>Glossina sp.</td><td>G</td><td>G | AA</td></tr>
<tr><td>P. serpens</td><td>18.4</td><td>47</td><td>Tomato</td><td>Phthia picta</td><td>G</td><td>G?</td></tr>
<tr><td>P. EM1</td><td>17.8</td><td>48</td><td>Euphorbia</td><td>Unknown insect</td><td>G</td><td>G?</td></tr>
<tr><td>P. francai</td><td>17.7</td><td>46</td><td>Cassava</td><td>Unknown insect</td><td>G</td><td>G?</td></tr>
<tr><td>P. HART1</td><td>18.1</td><td>46</td><td>Oil Palm</td><td>Unknown insect</td><td>G</td><td>G?</td></tr>
<tr><td>L. braziliensis</td><td>32.1</td><td>58</td><td>Mammals</td><td>Multiple sandflies</td><td>G | AA</td><td>AA | FA</td></tr>
<tr><td>L. tarentolae</td><td>31.1</td><td>59</td><td>Geko</td><td>Multiple sandflies</td><td>G | AA</td><td>AA | FA</td></tr>
<tr><td>L. mexicana</td><td>32.1</td><td>60</td><td>Mammals</td><td>Multiple sandflies</td><td>G | AA</td><td>AA | FA</td></tr>
<tr><td>L. major</td><td>32.9</td><td>60</td><td>Mammals</td><td>Multiple sandflies</td><td>G | AA</td><td>AA | FA</td></tr>
<tr><td>L. infantum</td><td>32.1</td><td>59</td><td>Mammals</td><td>Multiple sandflies</td><td>G | AA</td><td>AA | FA</td></tr>
<tr><td>L. donovani</td><td>32.4</td><td>59</td><td>Mammals</td><td>Multiple sandflies</td><td>G | AA</td><td>AA | FA</td></tr>
</table>

Phylogenetic trees and metabolic information for the parasites used in this study. Hosts and vectors are indicated where known. The major pathways used to generate ATP in each host are provided where G = glycolysis, AA = amino acid metabolism, AS = amino-sugar metabolism, FA = fatty acid metabolism. Genome size and GC content are also provided.

Supplemental Fig. S2.

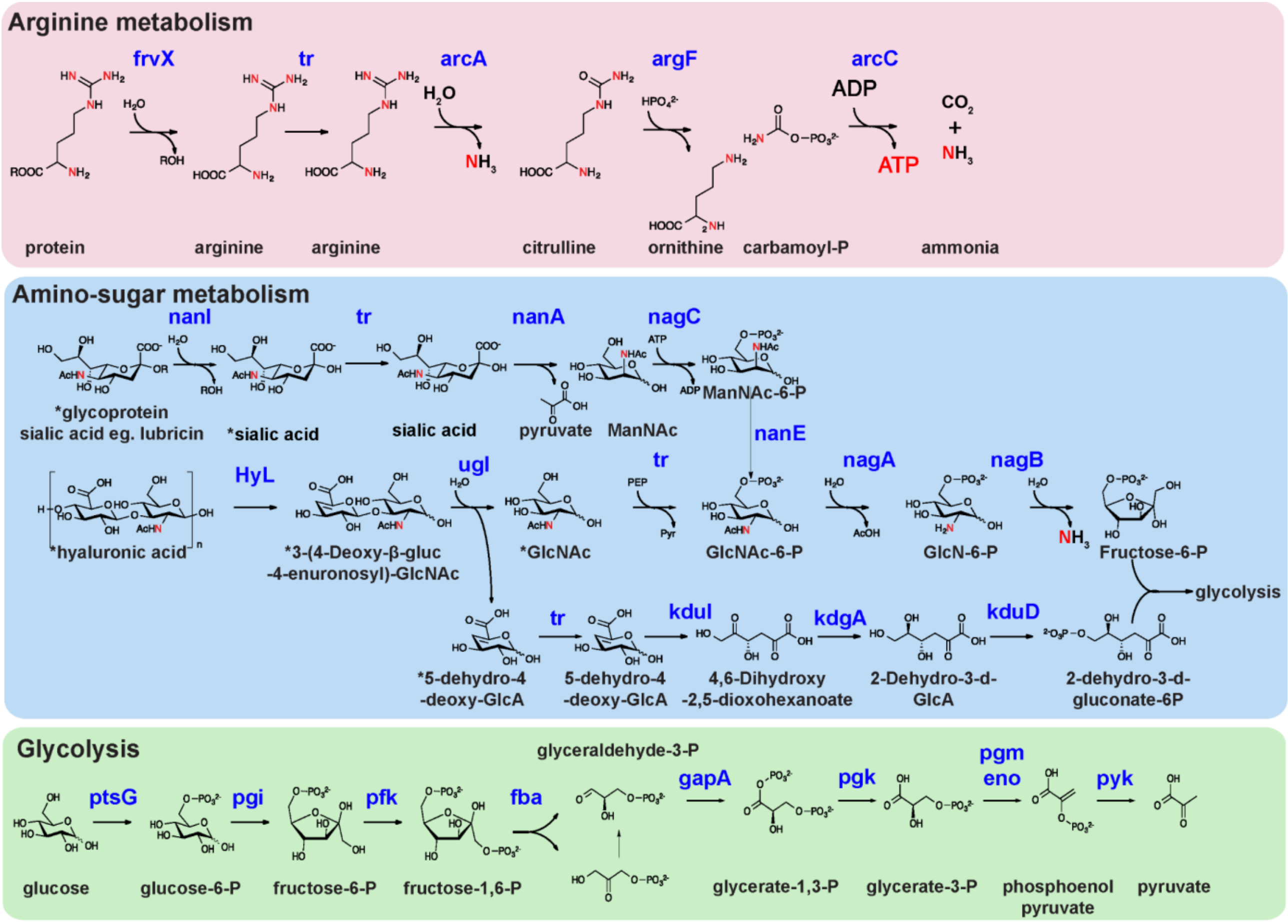

ATP generating metabolic pathways. Nitrogen atoms have been highlighted in red and required genes are indicated in blue. * denotes substrates that are extracellular. tr. is the abbreviation for transporter.

Supplemental Fig. S3.

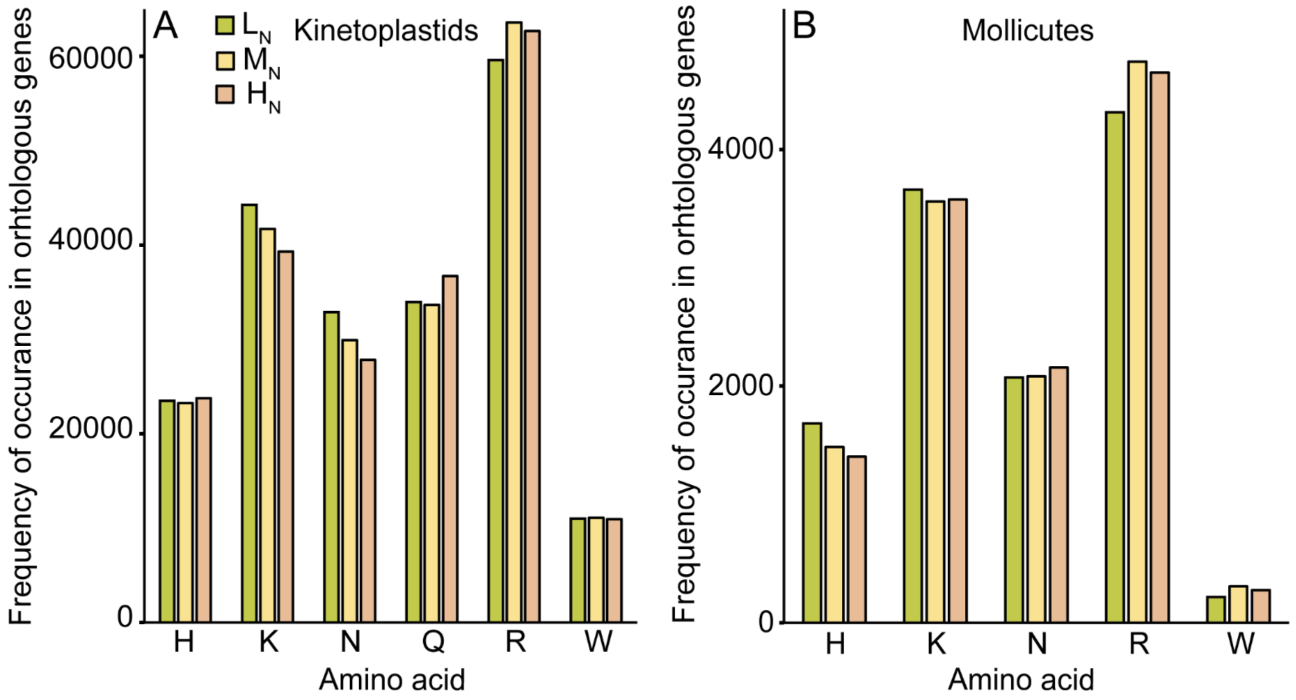

L_N parasites use the least amount of nitrogen in their amino acid side chains compared to M_N and H_N parasites. (*A*) Frequency of occurrence of amino acids with nitrogen in their side chains at orthologous sites in orthologous genes in the kinetoplastid parasites. This corresponds to a total number of nitrogen atoms in amino acids side chains of L_N = 347,789, M_N = 353,574 and H_N = 350,376. (*B*) Frequency of occurrence of amino acids with nitrogen in their side chains at orthologous sites in orthologous genes in the Mollicute parasites. This corresponds to a total number of nitrogen atoms in amino acids side chains of L_N = 11,948, M_N = 12,178 and H_N = 12,063. Glutamine (Q) is not considered for the Mollicutes as the M_N and H_N groups lack glutaminyl-tRNA synthetase (GlnS). Instead a non-discriminating glutamyl-tRNA synthetase (GltX) charges both $tRNA^{Glu}$ and $tRNA^{Gln}$ with Glu. This means use of Q between *Mycoplasma* (M_N and H_N species) and *Phytoplasma* (L_N species) is not comparable. For both the kinetoplastids and the Mollicutes, M_N parasites use more nitrogen in their side chains than H_N parasites. This can be explained by the reduced occurrence of arginine (R) in the H_N parasites, which is expected as these parasites metabolise arginine to generate energy. In both graphs the orthologous sites are the same as for the analysis in the section “Low nitrogen availability parasites have low nitrogen content sequences and vice-versa”.

Supplemental Fig. S4.

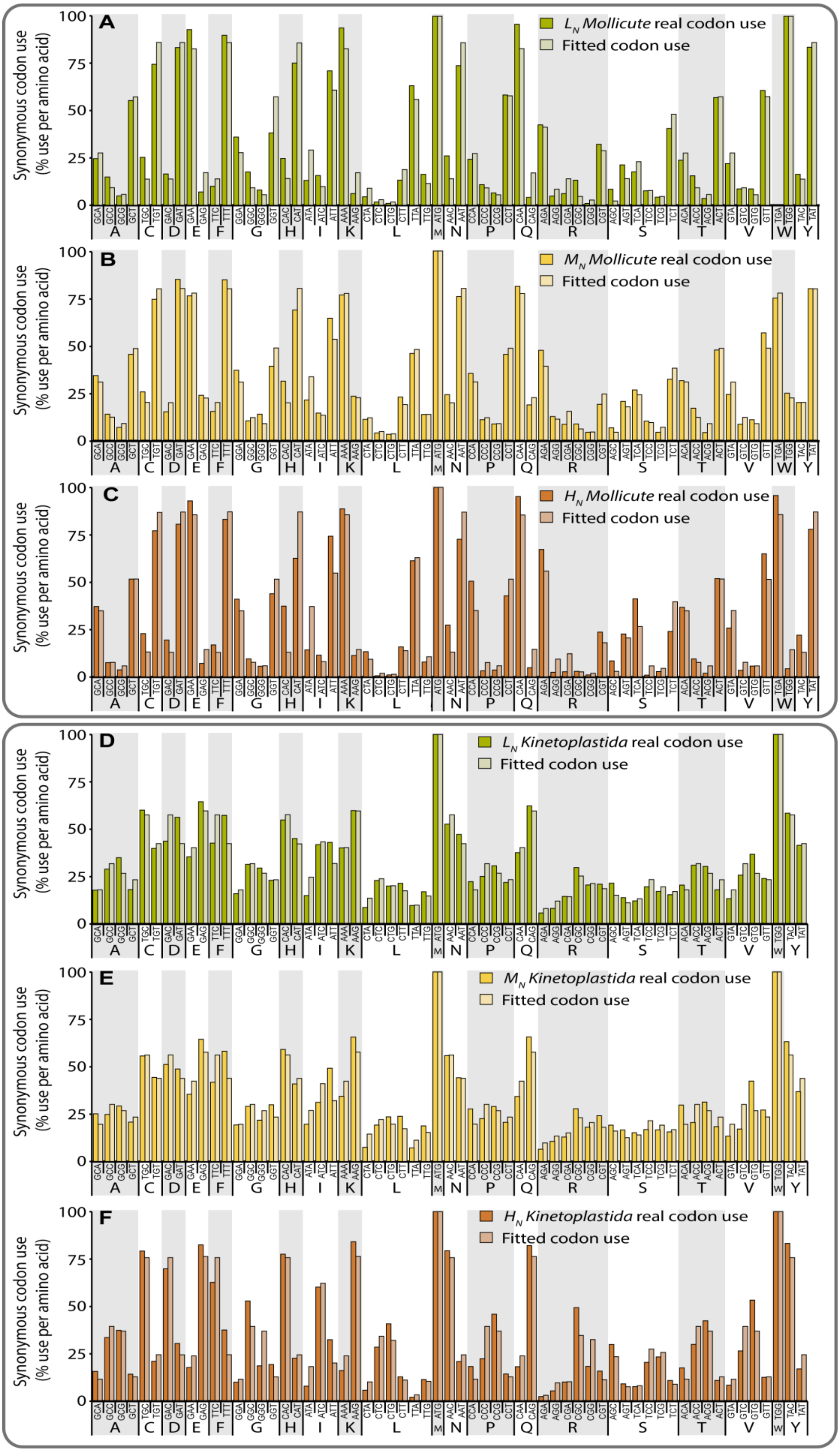

The model for synonymous codon use under the joint pressures of selection acting on nitrogen content and mutation bias fits real codon use with > 90% accuracy. Comparison of observed (dark) and fitted (light) synonymous codon use for (A) Mollicute L_N , $2N_g s$ = -0.24, *m* = 4.8 (B) Mollicute M_N, $2N_g s$ = -0.15, *m* = 3.5 (C) Mollicute H_N, $2N_g s$ = -0.13, *m* = 5.9 (D) Kinetoplastid L_N, $2N_g s$ = -0.09, *m* = 0.7 (E) Kinetoplastid M_N, $2N_g s$ = -0.06, *m* = 0.7 and (F) Kinetoplastid H_N $2N_g s$ = -0.03, *m* = 0.3 parasites.

Supplemental Fig. S5

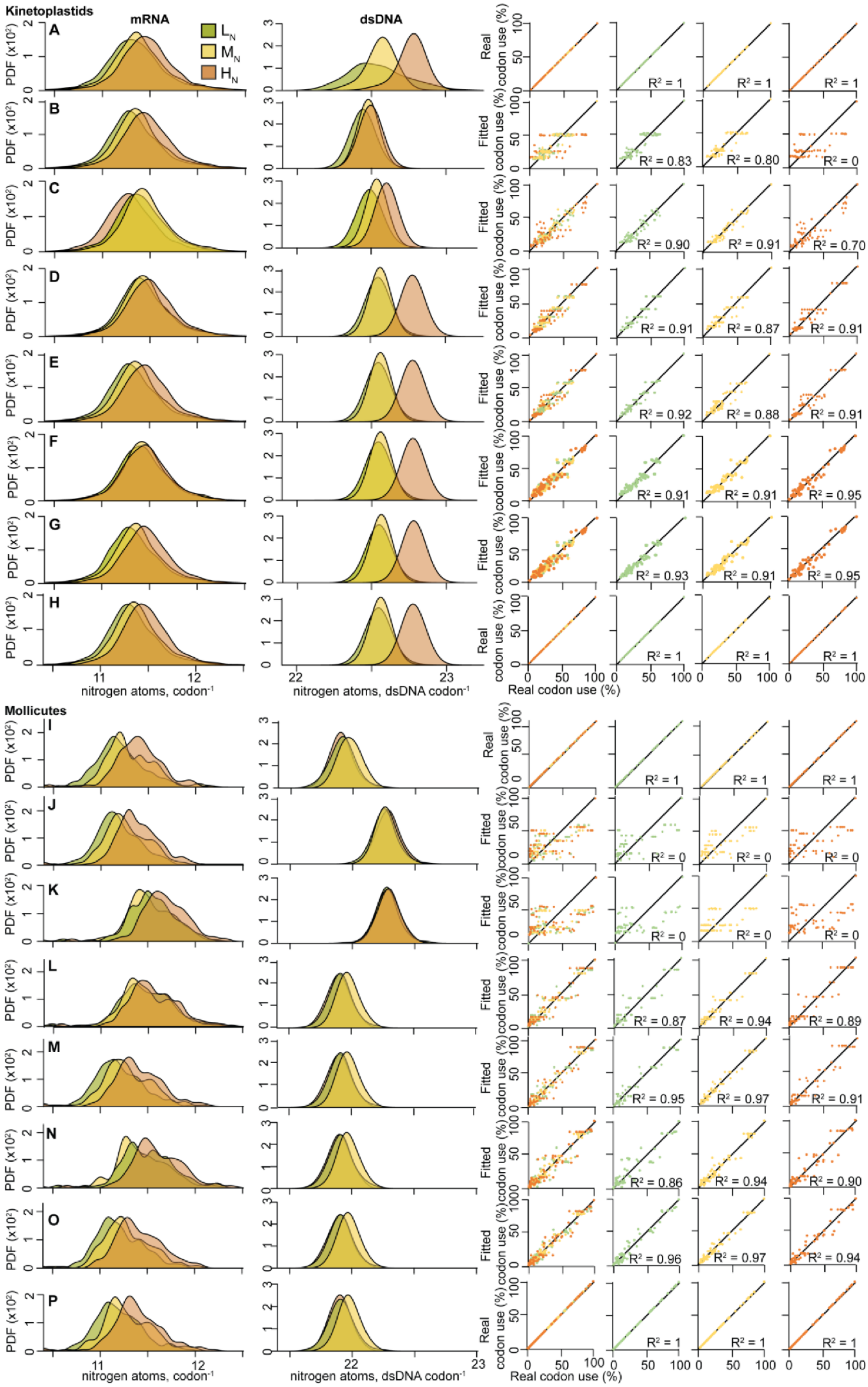

The model that considers both mutation bias and nitrogen content selection in combination provides a better fit than either parameter considered in isolation. (*A*) The average mRNA nitrogen content per codon for 3003 orthologous genes in the kinetoplastida. The average nitrogen content per double stranded codon for the same set of genes. Empirical codon use probabilities (expressed as %) plotted against themselves for all groups, L_N, M_N and H_N respectively. Analogous plots are shown in panels B-H for sequences simulated using fitted values for (*B*) selection acting on codon nitrogen content (Equation 2). (C) selection acting on translational efficiency (tAI) (Equation 10). (D) mutation bias acting on the GC content of the sequences (Equation 5). (E) both selection acting on codon nitrogen content and mutation bias (Equation 7) (F) both selection acting on translational efficiency and mutation bias (Product of equations 5 and 10). (G) all 3 parameters together i.e. Selection acting on codon nitrogen content, mutation bias and selection acting on translational efficiency (Equation 12) (H) empirical codon usage probabilities i.e. the 61 actual codon use frequencies. These simulated sequences produce symmetrical distributions with low variance that do not precisely recapitulate the data found in A. For the kinetoplastids (A-H) the best AIC values for L_N and M_N are 1992812 and 2011196 respectively for the 3 parameter model (G), for H_N it is 1756308 for the two parameter model that considers both translational efficiency and mutation bias. (*I – P)* As for plots A-H but for the Mollicutes. For the Mollicutes the best AIC values for L_N, M_N and H_N are 61440, 71126 and 59288 respectively for the 3 parameter model (O) .Y-axis is the probability density function (PDF) for the distributions.

Supplemental Fig. S6

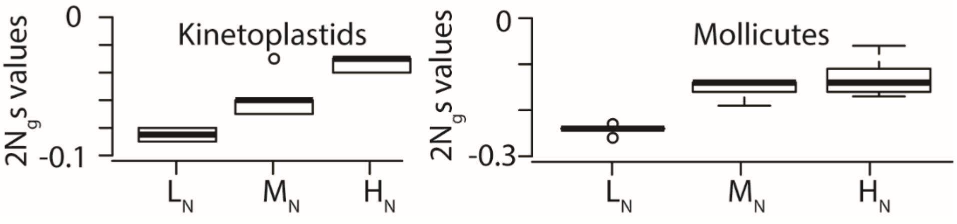

Boxplots showing distribution of $2N_gs$ values for individual species.

Supplemental Fig. S7

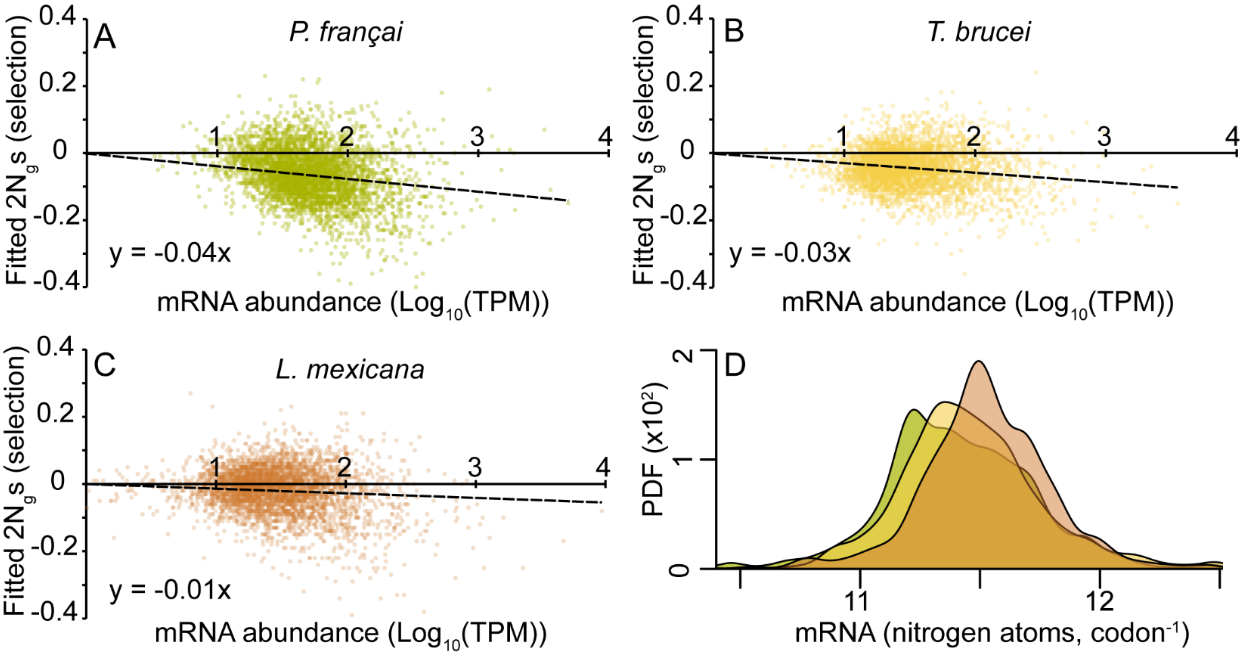

Gene expression negatively correlates with selection acting on mRNA nitrogen content. (*A*-*C*) Using the selection-mutation model, $2N_gs$ values were calculated for 4083 orthologous genes for one species from each of the L_N , M_N and H_N kinetoplastid groups (*P. françai, T. brucei* and *L. mexicana* respectively). Mutation bias values were fixed as constant at the overall value for each species (L_N = 0.61, M_N = 0.92 H_N = 0.34). These values were plotted against mRNA expression data from equivalent lifecycle stages (procyclic) and data points were set to an opacity value of 50% to help judge density. Linear regressions were fit to the data for each species. The slope of the L_N line was the most negative, M_N was intermediate and H_N was the closest to 0. This is consistent with our other results and shows that selection to minimise nitrogen in mRNA is strongest for the species that are the most nitrogen limited. ie a gene with a TPM value of 100 would be predicted to have a $2N_gs$ value of L_N = -0.08, M_N = -0.06 and H_N = -0.02. Two-tailed t-tests comparing the slopes showed that all of them were significantly different from one another ($p < 0.05$). R^2 = 0.07, 0.01 and 0.02 respectively. (*D*) Comparison of the nitrogen use of the 4083 orthologous genes found in all of all of *P. françai* (green), *T. brucei* (yellow) and *L. mexicana* (orange) taking expression into account. ie. If a gene had a TPM value of 10, it is represented in the distribution 10 times. All distributions are significantly different using a Wilcoxon Signed-Ranks ($p < 0.05$).

Supplemental Fig. S8

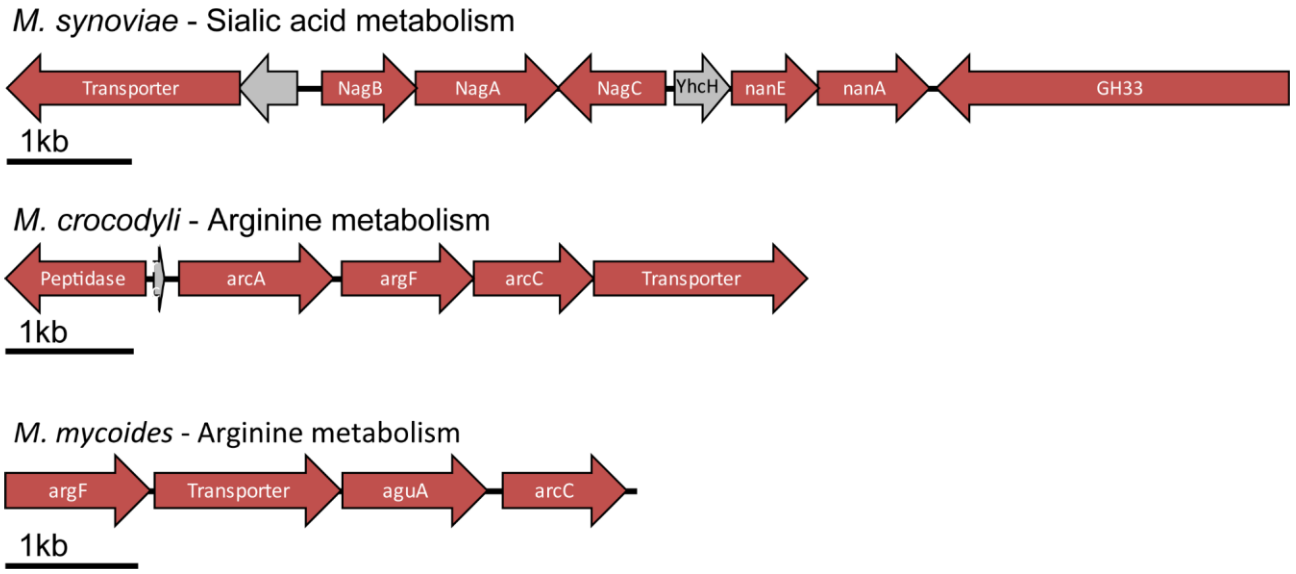

Gene clusters for nitrogen liberating metabolic pathways in H_N Mollicute parasites.

Supplemental Fig. S9

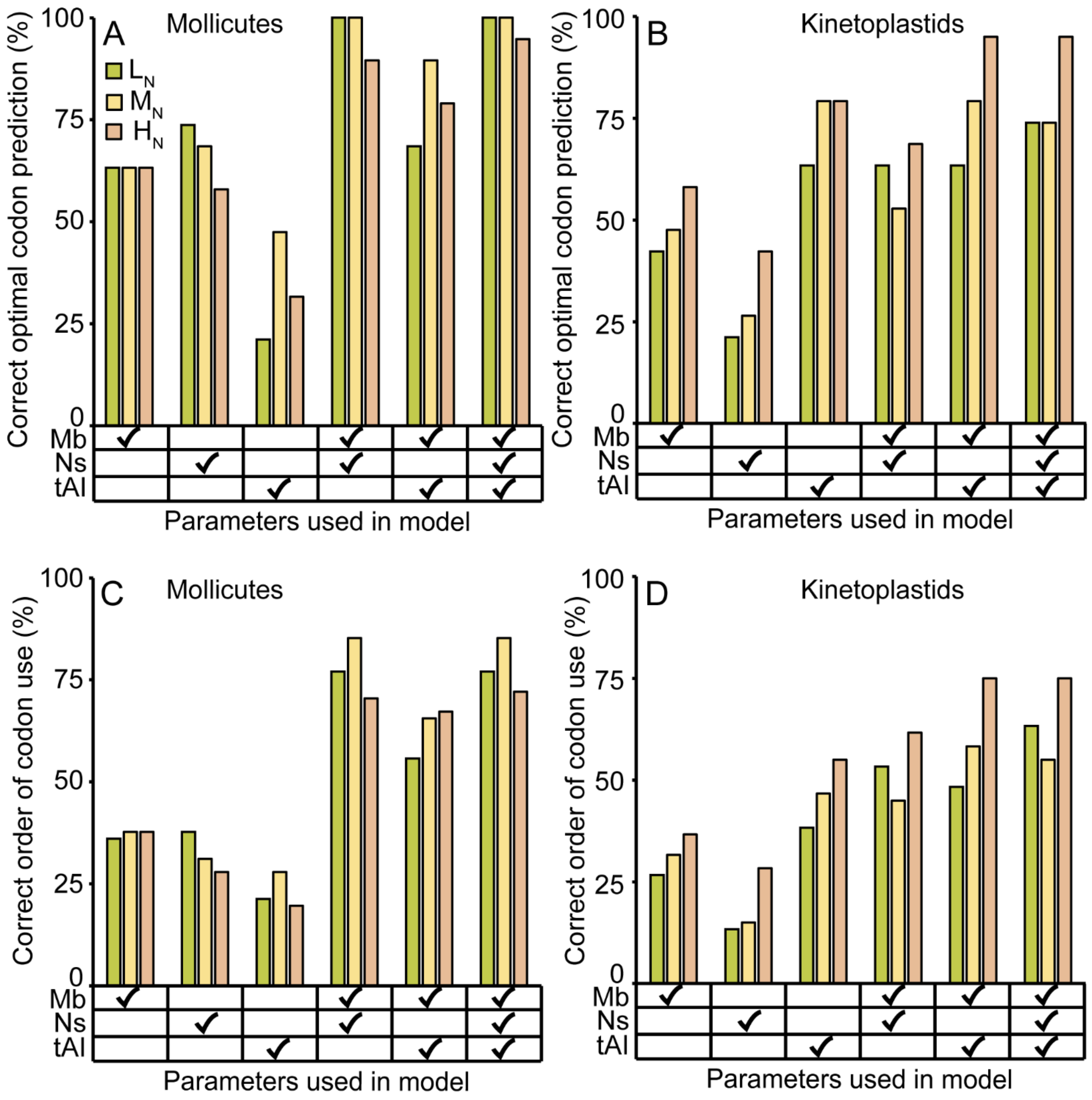

The model parameters which provide the best fit between observed and predicted codon use also provide the best percentage of correctly predicted optimal codons and correctly ordered codon use. (A) Percentage of correctly predicted optimal codons for Mollicute parasites using different parameter combinations. (B) As in (A) but for kinetoplastid parasites. (C) Percentage of correctly ordered relative codon use for different parameter combinations. (D) As in (C) but for kinetoplastid parasites. Mb = Mutation bias, Ns = selection acting on nitrogen content, tAI = selection acting on translational efficiency.

Supplemental Fig. S10

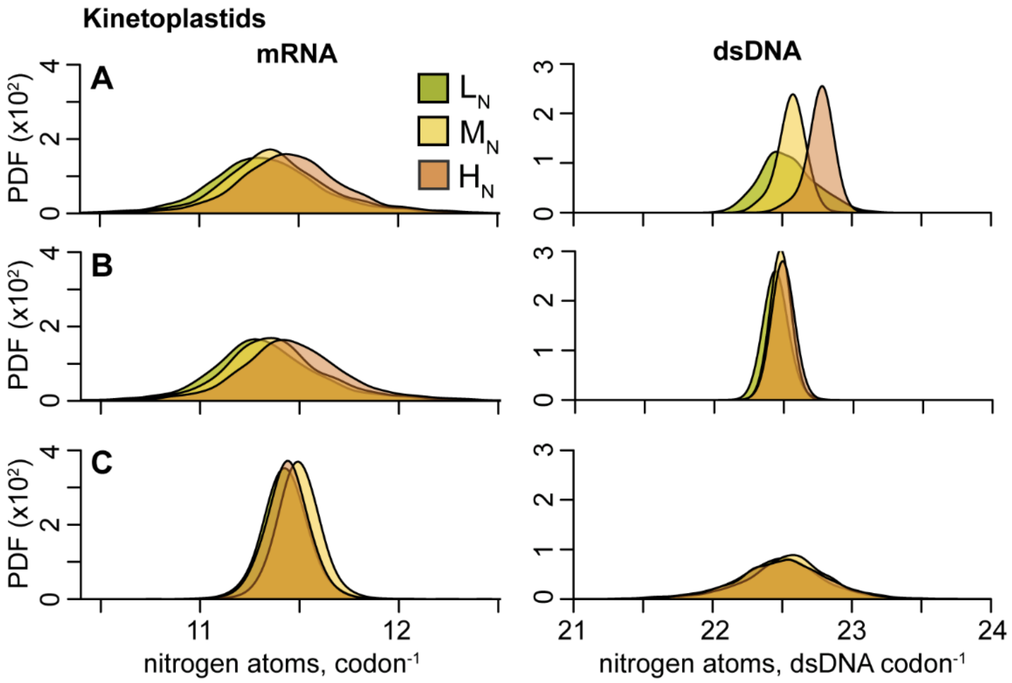

Example distribution of the model when run with shuffled codon nitrogen content. (*A*) Left, the average mRNA nitrogen content per codon for 3003 orthologous genes in the kinetoplastida (observed data). Right, the average nitrogen content per double stranded codon for the same set of genes. Analogous plots are shown in panels B and C for sequences simulated using fitted values for (*B*) selection acting on codon nitrogen content (Equation 2). (C) selection acting on codon nitrogen content where the nitrogen content of the codons has been shuffled (as described in the methods). Shuffled codon nitrogen cost (C) provides distributions that do not fit observed data (A) as well as distributions that use real nitrogen content (B). $2N_gs$ values for the shuffled example were L_N = 0.01, M_N = 0.02 and H_N = 0 compared to -0.06, -0.04 and 0.03 for the real nitrogen content. Thus when codon nitrogen content is shuffled, the model is unable to recapitulate the observed distribution of nitrogen content in gene sequences and the influence of the model parameter is reduced towards zero.
