# Supplementary material for: Dietary nitrogen alters codon bias and genome composition in parasitic microorganisms
Source: Genome Biol. 2016 Nov 15;17:226. doi: 10.1186/s13059-016-1087-9 (PMC5109750; doi:10.1186/s13059-016-1087-9)
Supplement: Additional file 2: — Supplemental information on parasite metabolism. (PDF 425 kb) [file 13059_2016_1087_MOESM2_ESM.pdf]

Kinetoplastida metabolic specialisation to host environment

The *Kinetoplastida* are a group of widespread single celled eukaryotic parasites that infect a broad range of hosts[1]. These parasites cause a global health burden on both humans and crops and have a dixenous lifestyle[2]. This means they have to adapt to two host environments and are exposed to conditions as disparate as tomato fruits and the insect mid-gut [2,3].

There is a plant-specific genus of *Kinetoplastida* called *Phytomonas*. These parasites have evolved multiple strategies to live in the carbohydrate-rich environment of plants. Firstly they utilize abundant plant sugars for their primary metabolism, feeding the glycolytic pathway via sucrose and trehalose metabolism[4]. Secondly *Phytomonas* have also been shown, despite depending on oxidative metabolism, to be able to survive without heme[5]. This is a unique metabolic adaptation as heme was thought to be a universally essential protein cofactor for fundamental cellular processes. Finally, unlike other kinetoplastids, *Phytomonas* do not undergo a metabolic switch from carbohydrate to amino acid metabolism when in their insect vectors[2]. This is probably possible due to the restricted feeding of their insect hosts on carbohydrate rich plant juices. This may have enabled the loss of a number of mitochondrial pathways such as the respiratory chain required for beta oxidation of fatty acids and the complete oxidation of amino acids[4].

Leishmania & Trypanosoma undergo metabolic shifts between hosts

Animal infective Kinetoplastids belonging to the genera *Leishmania* and *Trypanosoma* undergo metabolic shifts between their insect and animal hosts. For example *Trypanosoma brucei*, the causative agent of sleeping sickness in humans, undergoes significant morphological and metabolic adaptations between infecting the human bloodstream and the alimentary tract and salivary glands of tsetse flies, its insect vector[6]. *T. brucei* preferentially metabolises glucose, relying exclusively on glycolysis for ATP production in the bloodstream[7]. However if the glucose supply is limited or spent, such as in the tsetse fly mid-gut, *T. brucei* is able to switch metabolism to catabolism of amino acids such as proline[6].

Host metabolism is also known to affect parasite growth. For example proliferation of *T. cruzi,* the causative agent of human Chagas' disease has been shown to be linked to host metabolism[8]. In particular host nucleotide metabolism and energy production as well as fatty acid oxidation are key cellular processes that drive intracellular *T. cruzi* growth. Targeted changes to these host metabolic pathways modulated the parasite's ability to replicate, emphasising the flexibility of this intracellular pathogen to deal with fluctuating conditions in its host[8].

Similar to *Trypanosoma,* there is a metabolic shift between the insect and animal infective stages of *Leishmania mexicana*. The change between the *L. mexicana* promastigote stage (insect infective) and amastigote stage (infects the parasitophorous vaculole of white blood cells) has a concomitant change in metabolism. There is a shift in the cells from using glucose and protein as their carbon source to beta-oxidation of fatty acids and increased use of amino acids[9]. This shift reflects the changes in substrate availability as the parasitophorous vacuole, a major site of protein degradation, is thought to be generally rich in amino acids and poor in sugars[10]. In fact adding exogenous arginine or ornithine to infected macrophages stimulates the growth of intracellular amastigotes. This implies that amastigote replication may be limited by the availability of these amino acids.

Overview of mollicute metabolism

Mollicutes are a class of parasitic bacteria that have undergone dramatic genome reduction resulting in genomes smaller than 1.5 Mbp[11]. They are unable to synthesize purine and pyrimidine bases *do novo* and depend on import of nucleotide precursors from their host[12]. Furthermore the mechanism for the final phosphorylation step converting (d)NDPs to (d)NTPs remains elusive as there is no

nucleoside diphosphate kinase identified in Mollicutes[13]. Other enzymes have been proposed to substitute for this reaction but their relative contributions and comparative substrate specificities remain unclear[14]. Mollicutes not only exhibit highly reduced genomes but also limited metabolic capabilities that vary between even closely related species. For example fatty acid, amino acid and nucleotide biosynthesis are all disrupted, as is the tricarboxylic acid cycle and oxidative phosphorylation [15].

The plant infective genera of *Mollicute* bacteria, *Phytoplasma,* have adapted to a phytopathogenic lifestyle by evolving specialised sugar uptake and metabolism strategies[16,17]. These include loss of ATP-synthase genes, previously considered indispensable for life, and generation of ATP in a manner that is highly dependent on the glycolytic pathway[16]. *Candidatus P. mali* has gone one step further and jettisoned the genes for glycolysis and is proposed to generate ATP through catabolism of malate and maltose [17,18].

The closely related genera of *Mollicute* bacteria, *Mycoplasma*, are animal infective and colonise a range of tissues from the bloodstream to the synovial fluid of joints and the respiratory tract epithelium[19–21]. The environments that the *Mycoplasma* evolved to occupy have shaped the parasite's metabolism. For example *M. synoviae,* is a major poultry pathogen that infects the synovial fluid and respiratory tract[22]. Both cells on the upper respiratory tract and lubricin, a major component of the synovial fluid, have sialic acid residues. *M. synoviae* is able to cleave off, import and metabolise sialic acid in a pathway that liberates nitrogen in the form of ammonia and also feeds the glycolytic pathway for ATP generation. This pathway is not common in *Mycoplasma* and is an example of metabolic specialisation in adaptation to a specific host environment.

Host environment is not the only factor that determines the parasite's metabolism. Multiple metabolic strategies can be employed within the same ecological niche. For example three *Mollicute* bacteria, *M. hominis, M. genitalium* and *Ureaplasma parvum,* all reside in the same urogenital tract niche but have distinct energy generation strategies[23]. *M. genitalium* and *U. parvum* metabolise glucose and urea respectively. However *M. hominis,* which has the second smallest genome among self-replicating fee living organisms (only 537 coding sequences), has done away with ATP generation via glycolysis and instead generates ATP via arginine catabolism[23]. This provides a good opportunity to study of the impact of differential metabolic strategies on genome evolution, independent of environmental differences.

This type of metabolic tailoring can also be seen by comparative analysis of multiple *Mycoplasma* genomes. Differences in enzymatic pathways reflect the different genes required for carbohydrate, amino acid and nucleotide metabolism as well as host-pathogen interaction. *Mycoplasma* have different enzymatic compositions that reflect their host environment and are distinctive between species[24].

Interestingly, nitrogen metabolism in *Mollicutes* is perturbed. The canonical pathway for nitrogen assimilation, via the GS-GOGAT pathway or glutamate dehydrogenase (GDH), is not functional as the genes for glutamine synthetase (GS), glutamate synthase (GOGAT) and GDH are missing from *Mollicute* genomes[25]. Furthermore only the *Ureaplasma* appear to have the ammonium transporters *amt1* and *amt2* [25]. This indicates that there are unusual nitrogen utilisation strategies in these bacteria with the potential for novel pathway discovery.

1. Votýpka, J. *et al.* Probing into the diversity of trypanosomatid flagellates parasitizing insect hosts in South-West China reveals both endemism and global dispersal. *Mol. Phylogenet. Evol.*

54, 243–253 (2010).

2. Jaskowska, E., Butler, C., Preston, G. & Kelly, S. Phytomonas: Trypanosomatids Adapted to Plant Environments. *PLOS Pathog.* **11,** e1004484 (2015).

3. Jackson, A. P. Genome evolution in trypanosomatid parasites. *Parasitology* 1–17 (2014). doi:10.1017/S0031182014000894

4. Porcel, B. M. *et al.* The Streamlined Genome of Phytomonas spp. Relative to Human Pathogenic Kinetoplastids Reveals a Parasite Tailored for Plants. *PLoS Genet.* **10,** e1004007 (2014).

5. Ayala, F. J. & Luke, J. Aerobic kinetoplastid fl agellate Phytomonas does not require heme for viability. doi:10.1073/pnas.1201089109/-/DCSupplemental.www.pnas.org/cgi/doi/10.1073/pnas.1201089109

6. Mazet, M. *et al.* Revisiting the Central Metabolism of the Bloodstream Forms of Trypanosoma brucei: Production of Acetate in the Mitochondrion Is Essential for Parasite Viability. *PLoS Negl. Trop. Dis.* **7,** 1–14 (2013).

7. Spitznagel, D. *et al.* Alanine aminotransferase of Trypanosoma brucei- a key role in proline metabolism in procyclic life forms. *FEBS J.* **276,** 7187–7199 (2009).

8. Caradonna, K. L., Engel, J. C., Jacobi, D., Lee, C.-H. & Burleigh, B. a. Host metabolism regulates intracellular growth of Trypanosoma cruzi. *Cell Host Microbe* **13,** 108–17 (2013).

9. Fiebig, M., Kelly, S. & Gluenz, E. Comparative Life Cycle Transcriptomics Revises Leishmania mexicana Genome Annotation and Links a Chromosome Duplication with Parasitism of Vertebrates. *PLOS Pathog.* **11,** e1005186 (2015).

10. McConville, M. J. & Naderer, T. Metabolic pathways required for the intracellular survival of Leishmania. *Annu. Rev. Microbiol.* **65,** 543–61 (2011).

11. Grosjean, H. *et al.* Predicting the Minimal Translation Apparatus: Lessons from the Reductive Evolution of Mollicutes. *PLoS Genet.* **10,** (2014).

12. Mitchell, a. & Finch, L. R. Pathways of nucleotide biosynthesis in Mycoplasma mycoides subsp. mycoides. *J. Bacteriol.* **130,** 1047–1054 (1977).

13. Bizarro, C. V. & Schuck, D. C. Purine and pyrimidine nucleotide metabolism in Mollicutes. *Genet. Mol. Biol.* **30,** 190–201 (2007).

14. Pollack, J. D., Myers, M. a, Dandekar, T. & Herrmann, R. Suspected utility of enzymes with

multiple activities in the small genome Mycoplasma species: the replacement of the missing 'household' nucleoside diphosphate kinase gene and activity by glycolytic kinases. *OMICS* **6,** 247–258 (2002).

15. Chen, L.-L., Chung, W.-C., Lin, C.-P. & Kuo, C.-H. Comparative analysis of gene content evolution in phytoplasmas and mycoplasmas. *PLoS One* **7,** e34407 (2012).

16. Oshima, K. *et al.* Reductive evolution suggested from the complete genome sequence of a plant-pathogenic phytoplasma. *Nat. Genet.* **36,** 27–29 (2004).

17. Kube, M. *et al.* The linear chromosome of the plant-pathogenic mycoplasma 'Candidatus Phytoplasma mali'. *BMC Genomics* **9,** 306 (2008).

18. Kube, M., Mitrovic, J., Duduk, B., Rabus, R. & Seemüller, E. Current View on Phytoplasma Genomes and Encoded Metabolism. *Sci. World J.* **2012,** 1–25 (2012).

19. Waites, K. B., Waites, K. B., Talkington, D. F. & Talkington, D. F. Mycoplasma pneumoniae. *Society* **17,** 697–728 (2004).

20. Shu, H. W. *et al.* Genome sequence of the repetitive-sequence-rich Mycoplasma fermentans strain M64. *J. Bacteriol.* **193,** 4302–4303 (2011).

21. Nascimento, N. C. Do, Guimaraes, A. M. S., Santos, A. P., SanMiguel, P. J. & Messick, J. B. Complete genome sequence of Mycoplasma haemocanis strain Illinois. *J. Bacteriol.* **194,** 1605–1606 (2012).

22. T, D. D., I, D. Ben, Narat, M. & Oven, I. Phenotypic Characterization of Mycoplasma synoviae Induced Changes in the Metabolic and Sensitivity Profile of In Vitro Infected Chicken Chondrocytes. **2014,** (2014).

23. Pereyre, S. *et al.* Life on arginine for Mycoplasma hominis: Clues from its minimal genome and comparison with other human urogenital mycoplasmas. *PLoS Genet.* **5,** (2009).

24. Arraes, F. B. M. *et al.* Differential metabolism of Mycoplasma species as revealed by their genomes. *Genet. Mol. Biol.* **30,** 182–189 (2007).

25. Amon, J., Titgemeyer, F. & Burkovski, A. Common patterns - Unique features: Nitrogen metabolism and regulation in Gram-positive bacteria. *FEMS Microbiol. Rev.* **34,** 588–605 (2010).
